# Effects of Fat and Fatty Acids on the Formation of Autolysosomes in the Livers from Yellow Catfish *Pelteobagrus Fulvidraco*

**DOI:** 10.3390/genes10100751

**Published:** 2019-09-25

**Authors:** Li-Xiang Wu, Chuan-Chuan Wei, Shui-Bo Yang, Tao Zhao, Zhi Luo

**Affiliations:** 1 Laboratory of Molecular Nutrition for Aquatic Economic Animals, Fishery College, Huazhong Agricultural University, Wuhan 430070, China; wulixiang1234@126.com (L.-X.W.); yangshuibo123@webmail.hzau.edu.cn (S.-B.Y.); zhaotao2017@webmail.hzau.edu.cn (T.Z.); 2 Laboratory for Marine Fisheries Science and Food Production Processes, Qingdao National Laboratory for Marine Science and Technology, Qingdao 266237, China

**Keywords:** autolysosomes, fatty acids, molecular characterization, high-fat diet, fish

## Abstract

The autophagy-lysosome pathway, which involves many crucial genes and proteins, plays crucial roles in the maintenance of intracellular homeostasis by the degradation of damaged components. At present, some of these genes and proteins have been identified but their specific functions are largely unknown. This study was performed to clone and characterize the full-length cDNA sequences of nine key autolysosome-related genes (*vps11, vps16, vps18, vps33b, vps41, lamp1, mcoln1, ctsd1* and *tfeb*) from yellow catfish *Pelteobagrus fulvidraco*. The expression of these genes and the transcriptional responses to a high-fat diet and fatty acids (FAs) (palmitic acid (PA) and oleic acid (OA)) were investigated. The mRNAs of these genes could be detected in heart, liver, muscle, spleen, brain, mesenteric adipose tissue, intestine, kidney and ovary, but varied with the tissues. In the liver, the mRNA levels of the nine autolysosome-related genes were lower in fish fed a high-fat diet than those fed the control, indicating that a high-fat diet inhibited formation of autolysosomes. Palmitic acid (a saturated FA) significantly inhibited the formation of autolysosomes at 12 h, 24 h and 48 h incubation. In contrast, oleic acid (an unsaturated FA) significantly induced the formation of autolysosomes at 12 h, but inhibited them at 24 h. At 48 h, the effects of OA incubation on autolysosomes were OA concentration-dependent in primary hepatocytes of *P. fulvidraco*. The results of flow cytometry and laser confocal observations confirmed these results. PA and OA incubation also increased intracellular non-esterified fatty acid (NEFA) concentration at 12 h, 24 h and 48 h, and influenced mRNA levels of fatty acid binding protein (*fabp*) and fatty acid transport protein 4 (*fatp4*) which facilitate FA transport in primary hepatocytes of *P. fulvidraco*. The present study demonstrated the molecular characterization of the nine autolysosome-related genes and their transcriptional responses to fat and FAs in fish, which provides the basis for further exploring their regulatory mechanism in vertebrates.

## 1. Introduction

Autophagy is significantly conserved during evolution and plays important regulatory roles in the degradation of intracellular components through the lysosomes [1,2]. Lysosomes are acidic organelles responsible for the catabolism of damaged organelles and macromolecules following their fusion with autophagosomes [3]. Thus, the autophagy–lysosome pathway plays crucial roles in the maintenance of intracellular homeostasis by the degradation of damaged components. The important pathway involve many crucial genes and proteins, some of which have been identified but their specific functions are largely unknown. Among these genes and proteins, CORVET (class C core vacuole/ endosome tethering) and HOPS (homotypic fusion and protein sorting) belong to the members of the Vps-C complexes, regulate the membrane fusion and control membrane traffic [4,5]. HOPS controls the membrane fusion at the vacuolar lysosomes [5]. Accordingly, CORVET and HOPS play vital roles in the maintenance of the physiological integrity and functions of eukaryotic cells [6,7]. Class C Vps members include vacuolar protein sorting-associated protein (Vps) 11 (Vps11), Vps18, Vps16 and Vps33, which mediate the multiple pathways for vesicle transport [8,9]. Vps39 and Vps41 are two accessory subunits. They interact with the Vps-C core to form the large Class C Vps/HOPS complex [10]. Transcription factor EB (TFEB) modulates the expression of autophagy-related genes and mediates the formation of autophagosome and the degradation of lysosomes [11]. The members of the cathepsin family belong to the proteinase from the lysosomes [12]. Among these members, cathepsin D1 (CTSD1) plays an important role in the degradation of proteins mediated by lysosomes [13]. Lysosome-associated membrane glycoprotein1 (LAMP1) plays important roles in the auto (phago)-lysosomal fusion during the autophagic process [14]. Mucolipin-1 (MCOLN1) is one crucial lysosomal membrane protein and the calcium channel even involved in TFEB activation [15]. At present, the information of these genes and proteins has been investigated in many organisms, including *Drosophila*, *Caenorhabditis elegans* and mammals [16], suggesting that their functions were conserved among eukaryotes. However, in fish, studies involved in the sequence information of these genes have been very scarce. They have been reported in very limited numbers of fish, such as zebrafish *Danio rerio vps11, vps18* and *tfeb* [17,18,19], *ctsd1* in channel catfish [20], tilapia [21], sea bream [22]. Their mRNA tissue expression was also explored in several fish [21,22,23,24]. However, the molecular characterization and tissue distribution of *lamp1, mcoln1, vps41, vps16* and *vps33b* genes are still unknown in teleosts. Identification of these autolysosome genes was a key step for elucidating the important roles autophagy plays in fish.

Fat is one of the main nutrients in the diet and provides essential fatty acids and energy for fish. Adequate dietary fat levels promote the growth of fish. However, high fat levels can lead to excessive lipid deposition in the liver and induce the occurrence of many metabolic diseases which is mediated by lipotoxicity [25,26]. Fatty acid-induced hepatic lipotoxicity results in the occurrence of non-alcoholic fatty liver disease [25,27]. At present, evidence indicates that the autophagy–lysosome pathway is involved in the lipid-induced physiological and pathological responses [28]. Singh et al. (2009) pointed out that autophagy-mediated lipophagy helped to remove excess lipid droplets in hepatocytes [29]. However, how autophagy is modulated by fatty acids remains largely unknown [27]. Based on their structure, fatty acids are classified as unsaturated and saturated fatty acids, respectively. Palmitic acid (PA) and oleic acid (OA) belong to the saturated and unsaturated fatty acids, respectively. They are also the predominant FAs present in the diet and in serum [30]. Studies suggested that PA and OA had different effects on TG (triglyceride) accumulation and autophagy [31,32]. However, this has not been explored in detail. High-fat foods have been consumed in higher quantities in recent decades, which has led to the occurrence of obesity worldwide. Therefore, understanding the mechanism involved high fat-induced obesity becomes extremely important.

Fish are the biggest category of vertebrates in the world, but relevant studies of autophagy are very scarce [27]. Here, we identified the full-length cDNA sequences of nine important genes involved in autophagy–lysosome docking and fusion (*vps11, vps16, vps18, vps33b, vps41, lamp1, mcoln1, ctsd1* and *tfeb*) and explored their mRNA tissue expression in yellow catfish *P. fulvidraco*, an economic teleost in several Asian countries. Then, their mRNA expression for fish fed high-fat diets was also investigated, and the differential effects of PA and OA on mRNA levels of these genes and autophagy were understood.

## 2. Materials and Methods

Three experiments were performed. Experiment 1 was conducted to identify nine autolysosome-related genes, and study their mRNA tissue expression. Experiment 2 determined their expression in the liver of yellow catfish fed two lipid levels of diets. Experiment 3 explored the transcriptional responses of those genes and the formation of autolysosome in the hepatocytes of yellow catfish after PA and OA incubation, respectively. All experiments in the animals and cells followed the ethical guidelines of Huazhong Agricultural University (HZAU) and the manuscript conforms to the ARRIVE Guidelines for Reporting Animal Research. The experimental protocols were approved by the Ethical Committee of HZAU (identification code: Fish-2017-0219, Date: 28 September 2017).

### 2.1. Identification and Tissue Distribution of Gene Expression

Mixed sexes of yellow catfish *P. fulvidraco* (initial weight: 22.48 ± 3.15 g) were obtained from a farm in Wuhan (Hubei Province, China). Liver, spleen, muscle, brain, mesenteric fat, heart, intestine, kidney and ovary were collected from 12 similar-sized fish by using sterile scissors and pincers. They were frozen quickly in liquid nitrogen and kept at −80 °C for cloning and tissue expression analysis. In our study, tissue samples from 4 fish were mixed together and used as one biological replicate. The experimental procedures for RNA isolation and cDNA cloning were similar to those by Wei et al. [27]. Information of primers was shown in Supplementary Table S1. Their full-length cDNA sequences were obtained by the nested 3′ and 5′ RACE PCR (Rapid-Amplification of cDNA Ends PCR), and analyzed using the program EDITSEQ (DNA star) to search for the open reading frame (ORF). Standard genetic codes were used to translate them into amino acid sequences. We aligned these sequences, assessed the percentage of amino acid conservation by using Clustal-W tools, and analyzed their domains by the CDD (Conserved Domain Database) tool (http://www.ncbi.nlm.nih.gov/Structure/cdd/wrpsb.cgi). The neighbor-joining (NJ) method with MEGA 5.0 [33], was used to build the phylogenetic trees. The detailed protocols were described in our recent publication [27]. Bootstrap sampling was reiterated 1000 times.

### 2.2. Feed Preparation and Fish Culture

The feed formulation, culturing and management of *P. fulvidraco* were shown in our parallel study [34]. Briefly, two diets were formulated with dietary lipid levels at 11.34% and 15.41%, respectively. Fish oil and soybean oil (1:1, w/w) were used as the lipid sources. Dietary lipid levels were chosen based on our preliminary trial, and 11.34% and 15.41% of dietary lipid levels were considered to be adequate and excessive levels for *P. fulvidraco*, respectively. They were kept in the freezer at −20 °C until used. Each diet was assigned to three tanks in a completely randomized design, with 6 tanks for the experiment. Thirty uniform-sized fish (mean initial weight: 3.79 ± 0.16 g) were stocked in each fiberglass tank (300 L water volume), and were fed to apparent satiation twice daily. The experiment continued for 8 weeks.

### 2.3. In Vitro Studies

#### 2.3.1. Primary Hepatocytes Culture and Treatments

Primary hepatocytes were isolated and cultured from *P. fulvidraco*, based on the methods previously described [35,36]. They were incubated in a 28 °C incubator with 5% CO_2_ with OA and PA, respectively. Sampling occurred at 12 h, 24 h and 48 h, respectively. For each FA incubation, four treatments were chosen: control, 0.1 mM, 0.25 mM and 0.4 mM PA/OA addition. The concentration was based on our pilot trials and these are in public publications [37,38]. Each treatment had three replicates.

#### 2.3.2. Cell Viability and Non-Esterified Fatty Acid (NEFA) Concentrations Detection

MTT (3-(4, 5-dimethylthiazol-2-yl)-2,5 diphenyl tetrazolium bromide)was used to test the cell viability after Wu et al. [35]. Non-esterified fatty acid (NEFA) concentrations were determined by the commercial kits (Nanjing Jiancheng Bio-engineering Institute, China) according to the manufacturer’s instructions.

#### 2.3.3. Flow Cytometry and the Laser Confocal Microscope for Detecting the Formation of Autolysosomes

Cells were cultured in 12-well plates and treated with the corresponding reagents for the designed times, respectively. Thereafter, cells were stained with LysoTracker^TM^ Red DND-99 (L7528; Thermo Fisher Scientific), and FA-induced changes of autolysosomes activity were detected by flow cytometry [29]. In addition, hepatocytes of yellow catfish were co-stained with Hoechst (blue) and Lyso Tracker (red). The intensity of fluorescence was visualized by a laser-scanning confocal microscope (Leica Microsystems, Wetzlar, Germany), and quantified on a CytoFLEX Flow Cytometer (Beckman Coulter, WA, USA) and data analysis was performed using FlowJo VX software (Beckman Coulter, WA, USA).

#### 2.3.4. Real-Time Quantitative Polymerase Chain Reaction (qPCR)

The mRNA expression of nine genes were measured by the quantitative polymerase chain reaction (qPCR) methods described in our recent publications [27,34]. In order to test their transcriptional stability, we selected ten housekeeping genes (*18s rRNA*, *β-actin*, *rpl7*, *tuba*, *b2m*, *elfa*, *gapdh*, *tbp*, *hprt* and *ubce*). Supplementary Table S2 shows the primer sequences of all these genes. The 2^-ΔΔCt^ method was used to calculate mRNA expression after normalizing to the geometric mean of the best combination of two genes, which was determined by geNorm. The present study found that *gapdh* and *rpl7* (M = 0.1295), *b2m* and *18s rRNA* (M = 0.1152) showed the most stable expression in mRNA tissue expression and feeding experiments, and that *b2m* and *rpl7* (M = 0.2456), *gapdh* and *18s rRNA* (M = 0.1887, M = 0.3412), *tuba* and *β-actin* (M = 0.1550), *b2m* and *gapdh* (M = 0.3417, M=0.1473) were the most stable in the in vitro studies.

### 2.4. Statistical Analysis

The data were presented as means ± standard error of the mean (SEM). Before statistical analysis, the Kolmogornov–Smirnov test was used to analyze their normality of distribution. Then, one-way analysis of variance (ANOVA) and Duncan′s multiple range tests were used to analyze the significant differences of the data among the treatments. Student’s *t*-tests were used to compare the differences between two groups. SPSS 19.0 (IBM, Armonk, NY, USA) was used to perform these analyses. Significant levels were set at *p* < 0.05.

## 3. Results

### 3.1. Molecular Characterization

We obtained the full-length cDNA sequences of nine autolysosome-related genes (*vps11, vps16, vps18, vps33b, vps41, lamp1, mcoln1, ctsd1* and *tfeb*) from *P. fulvidraco* (Table 1). They ranged from 1496 bp to 3379 bp, with open reading frame (ORF) of 1245–2979 bp, respectively, encoding the protein of 396–992 amino acids (aa). We aligned these predicted polypeptide sequences and listed the amino acid sequences of nine autophagy-related genes among different species in Table 2. The structures of *P. fulvidraco vps11, vps16, vps18, vps33b, vps41, lamp1, mcoln1, ctsd1* and *tfeb* exhibited 27.61%–96.02% amino acid sequence identities compared to those from other species.

Protein sequence analysis predicted that VPS11 was a soluble protein and had a Clathrin repeat domain, a RING-H2 domain, RING-H2 finger motif and Zn binding site (Supplementary Figure S1). The *P. fulvidraco* VPS16 consisted of an N-terminal domain, and a C-terminal domain with coiled coil domain (Supplementary Figure S2). The *P. fulvidraco* VPS18 structure was similar to those in mammals, and included the Clathrin repeat domain and the RING-H2 finger domain with RING-H2 finger motif and Zn binding site (Supplementary Figure S3). The *P. fulvidraco* VPS33B was cytosolic and contained a Sec1 domain (Supplementary Figure S4). *P. fulvidraco* VPS41 included the Clathrin repeat domain, RING-H2 domain with RING-H2 finger motif and several Zn binding sites (Supplementary Figure S5). *P. fulvidraco* LAMP1 included the conservative luminal domain, transmembrane helix domain, C-terminal tail and one hinge region (Supplementary Figure S6). We also found the conserved catalytic cysteine residue and N-glycosylation site in *P. fulvidraco* LAMP1 (Supplementary Figure S6). *P. fulvidraco* MCOLN1 showed similar architecture with mammals, containing six transmembrane domains, C-terminal cytoplasmic tails and channel pore (Supplementary Figure S7). CTSD1 from *P. fulvidraco* was composed of propeptide, signal peptide and N-glycosylation site, KKXX-like motif (Supplementary Figure S8). The conserved catalytic cysteine residues were also found in *P. fulvidraco* CTSD1. TFEB included conserved helix-loop-helix domain, the leucine zipper, glutamine-rich basic domain (QB), charged helical domain, consensus MAP kinase phosphorylation site (MAPK) and some DNA binding sites (Supplementary Figure S9). The analysis of the phylogenetic tree further verified the genes and visualised the inferred evolutionary relationship (Supplementary Figures S10–S18).

### 3.2. Tissue Distribution of Gene Expression

**Figure 1:**
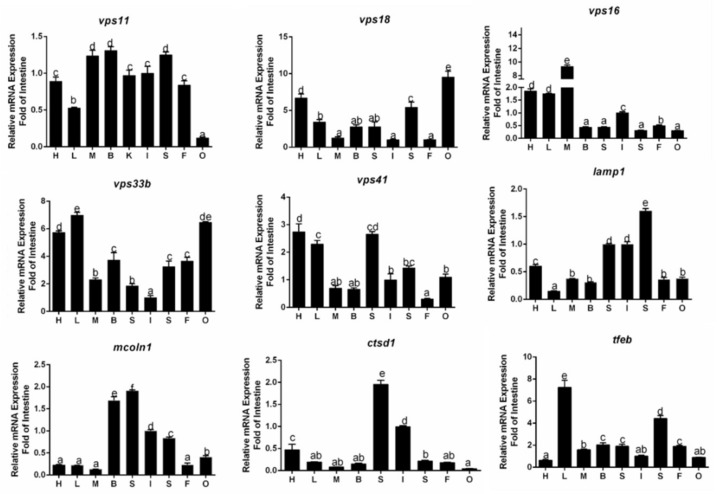
Real-time quantitative polymerase chain reaction (qPCR) analysis for tissue distribution of gene expression across the heart (H), liver (L), muscle (M), spleen (S), brain (B), mesenteric fat (F), intestine (I), kidney (K) and ovary (O) of *P. fulvidraco*. Bars that share different letters indicate significant differences among the tissues (*p* < 0.05).

mRNAs of these nine genes were widely detected in all sampled tissues, but their abundances differed (Figure 1). The *vps11* mRNA level was highest in the brain, muscle and spleen, followed by intestine, kidney, fat, heart, and liver, and lowest in the ovary (Figure 1). The *vps16* mRNA was predominant in the muscle, then in the heart, liver and intestine, and lowest in the fat, ovary, brain, kidney and spleen. The *vps18* mRNA was predominant in the ovary, then in heart, spleen, liver, brain and kidney, and lowest in muscle, intestine and fat. The *vps33b* had the highest expression in the liver and ovary, then in the heart, brain, fat, spleen, muscle and kidney, and lowest in the intestine. The *vps41* mRNA was highest in the heart and kidney, then in the liver, spleen, ovary, intestine, muscle and brain, and lowest in fat. The *lamp1* mRNA was predominant in the spleen, then in the intestine, kidney, heart, muscle, brain, fat and ovary, and lowest in the liver. The *mcoln1* mRNA was highest in the kidney, then in the brain, intestine, spleen and ovary, and lowest in the heart, fat, liver and muscle. The mRNA expression of *ctsd1* was predominant in the kidney, then in the intestine, heart, spleen, mesenteric fat, liver, brain and muscle, and lowest in the ovary. *tfeb* mRNA abundances were the highest in the liver, then in the spleen, brain, kidney, mesenteric fat, muscle, ovary and intestine, and lowest in the heart.

### 3.3. Transcriptional Responses of Nine Autolysosome-Related Genes by High-Fat Diet

**Figure 2:**
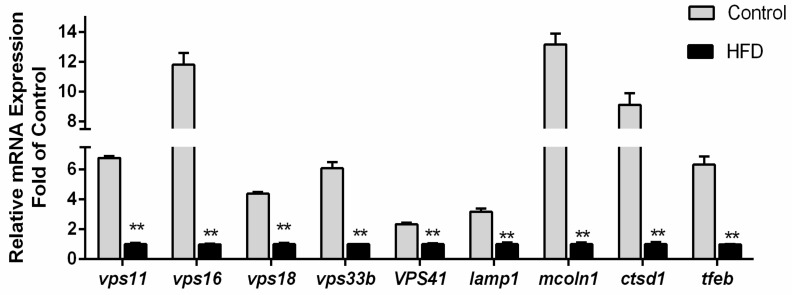
Effect of dietary fat levels on hepatic mRNA level of autolysosomes-related genes in *P. fulvidraco*. ^a,b,c^ Values are mean ± standard error of the mean (SEM), n = 3 (replicates of 3 fish); Asterisks indicate significant differences between two groups within the same gene (*p* < 0.05). CD = Control diet: HFD = High-fat diet.

Figure 2 showed the effects of a high-fat diet on the mRNA levels of the nine autolysosomes-related genes in the liver of yellow catfish. The mRNA levels of all these nine genes (*vps11, vps16, vps18, vps33b, vps41, lamp1, mcoln1, ctsd1* and *tfeb*) were significantly down-regulated (*p* < 0.05) by high-fat diets.

### 3.4. Effects of Palmitic Acid (PA) Incubations on the Formation of Autolysosomes

**Figure 3:**
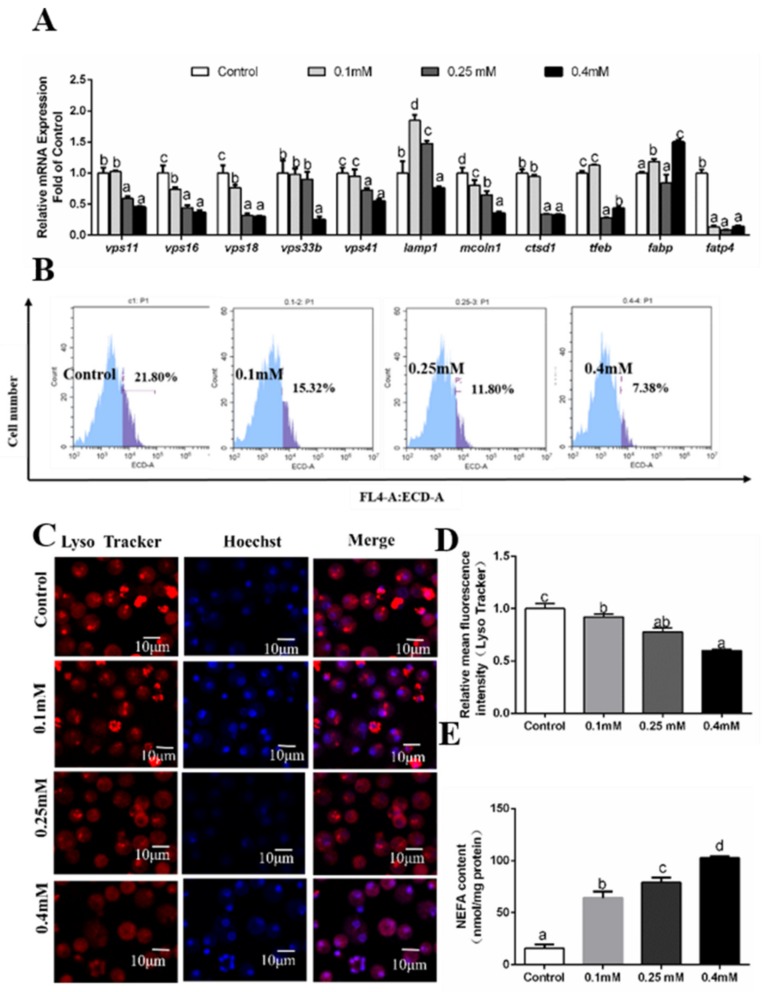
Effects of 12 h PA incubation on autophagosomes in hepatocytes of *P. fulvidraco.* (**A**) Effects of 12 h palmitic acid (PA) incubation on the mRNA levels of autolysosomes-related genes; data (mean ± SEM, n = 3) were expressed relative to expression of housekeeping genes (*b2m* and *rpl7* (M = 0.2456)). (**B**) The effect of 12h PA incubation on intracelluar autolysosomes demonstrated by flow cytometry analysis of the presence of Lyso Tracker-stained intracellular autolysosomes. (**C**) Representative confocal microscopy image of hepatocytes co-stained with Hoechest (blue) and Lyso Tracker (red) after 12 h PA incubation. (**D**) The effect of 12h PA incubation on intracelluar autolysosomes demonstrated by flow cytometric analysis of red (FL4) mean fluorescence intensity. (**E**) Effects of 12h PA incubation on non-esterified fatty acid (NEFA) content. Letters (*a–d*) denote significance, *p* < 0.05.

The MTT assay showed that PA concentrations of not more than 0.5 mM had no adverse influence on the viability of hepatocytes, which were chosen for the experiments. Among four PA groups, at 12 h, mRNA levels of *vps11, vps16, vps33b, vps41, mcoln1* and *ctsd1* were the highest for the 0.1 mM group and lowest for the 0.4mM PA group (Figure 3A). High PA concentrations (more than 0.25 mM) significantly reduced *vps18* and *tfeb* mRNA levels. Among four PA treatments, the *lamp1* mRNA expression was the lowest for 0.4 mM PA group (Figure 3A). The mRNA levels of *fabp* and *fatp4* were very variable and not related to PA concentrations. NEFA contents increased with increasing PA concentrations (Figure 3E). We also used LysoTracker Red staining for detecting the formation of autolysosomes. Flow cytometry results showed that PA addition significantly inhibited the formation of autolysosomes (Figure 3B,D). The results of laser confocal microscopy confirmed that autolysosomes were significantly inhibited after 12 h PA incubation (Figure 3C).

**Figure 4:**
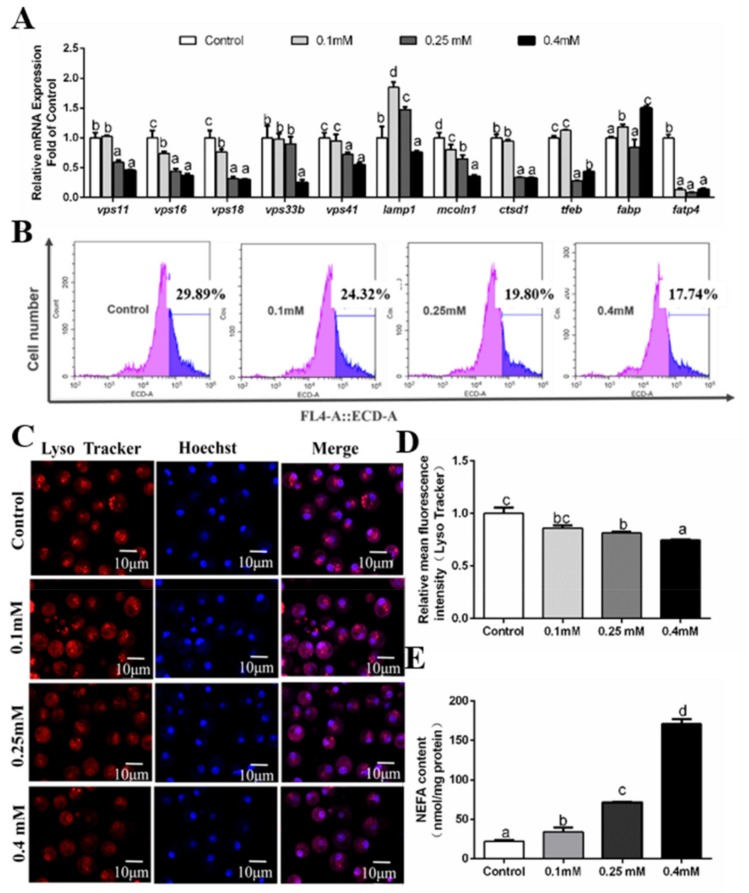
Effects of 24 h PA incubation on autophagosomes in hepatocytes of *P. fulvidraco.* (**A**) Effects of 24 h PA incubation on the mRNA levels of autolysosomes related genes; data (mean ± SEM, n = 3) were expressed relative to expression of housekeeping genes (*gapdh* and *18s rRNA* (M = 0.1887)). (**B**) The effect of 24 h PA incubation on intracelluar autolysosomes demonstrated by flow cytometry analysis of the presence of Lyso Tracker-stained intracellular autolysosomes. (**C**) Representative confocal microscopy image of hepatocytes co-stained with Hoechest (blue) and Lyso Tracker (red) after 2 4h PA incubation. (**D**) The effect of 24 h PA incubation on intracelluar autolysosomes demonstrated by flow cytometric analysis of red (FL4) mean fluorescence intensity. (**E**) Effects of 24 h PA incubation on NEFA content. Letters (*a–d*) denote significance, *p* < 0.05.

At 24 h, mRNA expression of *vps11* and *ctsd1* was predominant for the control and 0.1 mM PA group and lowest for other two groups (Figure 4A). The mRNA levels of *vps16, vps18, vps33b, vps41* and *mccoln1* tended to decline with increasing PA concentrations. The *lamp1* mRNAs were predominant for the 0.1 mM PA group and low for the 0.4 mM PA group. *tfeb* mRNAs were the lowest for the 0.25 mM PA group and highest for the 0.1 mM PA group and the control. *fabp* mRNAs were observed to be the highest in the 0.4 mM PA group and lowest in the control and 0.25 mM PA group (Figure 3C). PA addition in the medium reduced *fatp4* mRNA level (Figure 4A). NEFA contents increased with increasing PA concentrations (Figure 4E). We also used LysoTracker Red staining for detecting the formation of autolysosomes. Flow cytometry results showed that PA addition significantly inhibited the formation of autolysosomes (Figure 4B,D). The results of laser confocal microscopy confirmed that autolysosomes were significantly inhibited after 24 h PA incubation (Figure 4C).

**Figure 5:**
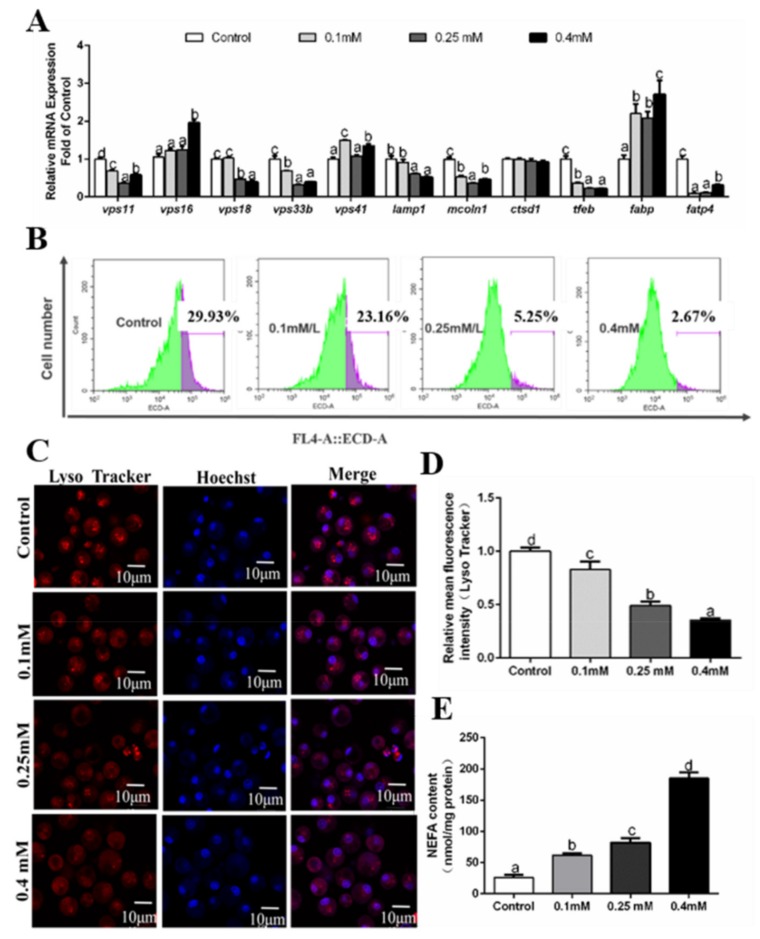
Effects of 48 h PA incubation on autophagosomes in hepatocytes of *P. fulvidraco.* (**A**) The effect of 48 h PA incubation on the mRNA levels of autolysosome-related genes; data (mean ± SEM, n = 3) were expressed relative to expression of housekeeping genes (*b2m* and *gapdh* (M = 0.1550)). (**B**) The effect of 48 h PA incubation on intracelluar autolysosomes demonstrated by flow cytometry analysis of the presence of Lyso Tracker-stained intracellular autolysosomes. (**C**) Representative confocal microscopy image of hepatocytes co-stained with Hoechest (blue) and Lyso Tracker (red) after 48 h PA incubation. (**D**) The effect of 48 h PA incubation on intracelluar autolysosomes demonstrated by flow cytometric analysis of red (FL4) mean fluorescence intensity. (**E**) Effects of 48 h PA incubation on NEFA content. Letters (*a–d*) denote significance, *p* < 0.05.

At 48 h, compared to the control, PA reduced mRNA abundances of *vps11, vps33b* and *tfeb* (Figure 5A), but tended to upregulate the *vps16* mRNAs. The mRNA expression of *vps18* and *lamp1* was higher in the control and 0.1 mM PA group than those in 0.25 mM and 0.40 mM PA groups. The *vps41* mRNA level was very variable and not related to PA incubation. The *ctsd1* mRNA level showed no significant differences among the four groups. PA addition significantly up-regulated *fabp* mRNA level but down-regulated *fatp4* mRNA level (Figure 5A). NEFA content increased with increasing PA incubation (Figure 5E). The flow cytometry analysis after Lyso Tracker staining indicated that 48 h PA incubation significantly reduced the formation of autolysosomes (Figure 5B,D). The laser confocal observation indicated that PA treatment significantly inhibited the formation of autolysosomes (Figure 5C).

### 3.5. Effects of Oleic Acid (OA) Incubations on the Formation of Autolysosomes

**Figure 6:**
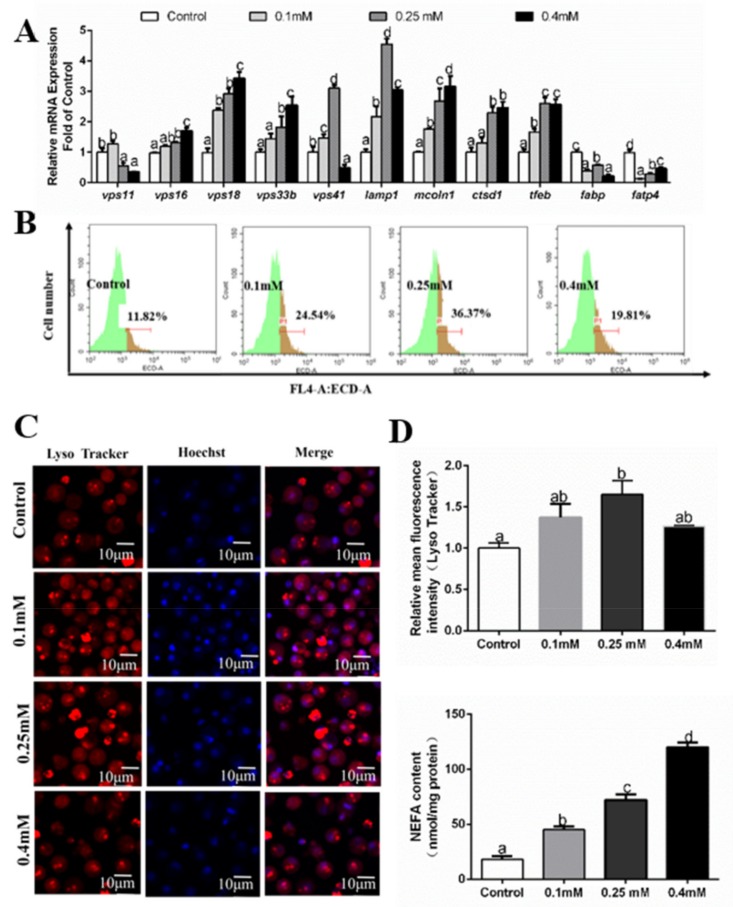
Effects of 12 h oleic acid (OA) incubation on autophagosomes in hepatocytes of *P. fulvidraco.* (**A**) Effects of 12 h OA incubation on the mRNA levels of autolysosome-related genes; data (mean ± SEM, n = 3) were expressed relative to expression of housekeeping genes (*b2m* and *rpl7* (M = 0.2456)). (**B**) The effect of 12 h OA incubation on intracelluar autolysosomes demonstrated by flow cytometry analysis of the presence of Lyso Tracker-stained intracellular autolysosomes. (**C**) Representative confocal microscopy image of hepatocytes co-stained with Hoechest (blue) and Lyso Tracker (red) after 12 h OA incubation. (**D**) The effect of 12 h OA incubation on intracelluar autolysosomes demonstrated by flow cytometric analysis of red (FL4) mean fluorescence intensity. (**E**) Effects of 12 h OA incubation on NEFA content. Letters (*a–d*) denote significance, *p* < 0.05.

At 12 h, compared to the control, OA incubation tended to increase the mRNA expression of *vps16, vps18, vps33b, lamp1, mcoln1, ctsd1* and *tfeb* (Figure 6A). However, *vps11* mRNA level was higher in the control and 0.1 mM OA group than those in 0.25 mM and 0.4 mM groups. *vps41* mRNAs were predominant in the 0.25 mM OA group and lowest in the 0.4 mM group. Compared to the control, OA addition markedly reduced mRNA expression of *fatp4* and *fabp*. Intracellular NEFA concentration increased with increasing OA concentration (Figure 6E). Moreover, we used flow cytometry and laser confocal microscopy to detect the OA-induced changes of autophagosomes, and results showed that OA incubation significantly inhibited the formation of autolysosomes (Figure 6B–D).

**Figure 7:**
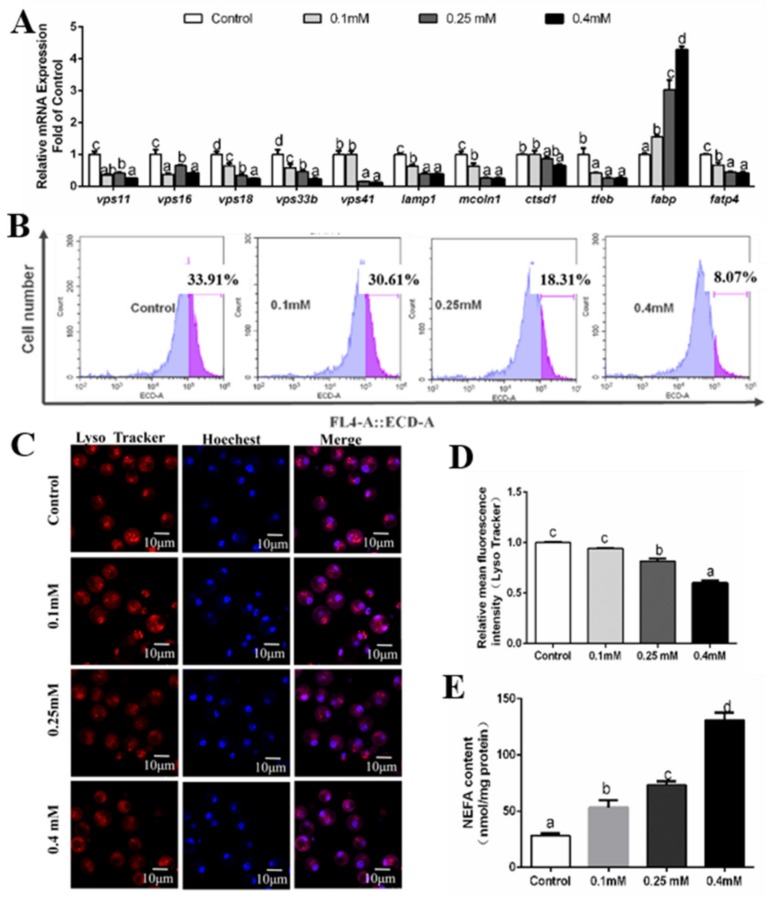
Effects of 24 h OA incubation on autophagosomes in hepatocytes of *P. fulvidraco.* (**A**) Effects of 24 h OA incubation on the mRNA levels of autolysosomes related genes; data (mean ± SEM, n = 3) were expressed relative to expression of housekeeping genes (*gapdh* and *18s rRNA* (M = 0.1887)). (**B**) The effect of 24 h OA incubation on intracelluar autolysosomes demonstrated by flow cytometry analysis of the presence of Lyso Tracker-stained intracellular autolysosomes. (**C**) Representative confocal microscopy image of hepatocytes co-stained with Hoechest (blue) and Lyso Tracker (red) after 24 h OA incubation. (**D**) The effect of 24 h OA incubation on intracelluar autolysosomes demonstrated by flow cytometric analysis of red (FL4) mean fluorescence intensity. (**E**) Effects of 24 h OA incubation on NEFA content. Letters (*a–d*) denote significance, *p* < 0.05.

At 24 h, compared to the control, OA addition tended to decline mRNA levels of *vps11, vps16, vps18, vps33b, lamp1, mcoln1* and *tfeb* (Figure 7A). *vps41* and *ctsd1* mRNA levels were higher in the control and 0.1 mM OA group than those in other two groups. OA addition increased *fabp* mRNA expression but reduced *fatp4* mRNA level (Figure 7A). Intracellular NEFA content increased with increasing OA concentration (Figure 7E). Then, we used flow cytometry and laser confocal microscopy to detect the OA-induced changes of autophagosomes, and our results showed that OA incubation significantly inhibited the formation of autolysosomes (Figure 7B–D).

**Figure 8:**
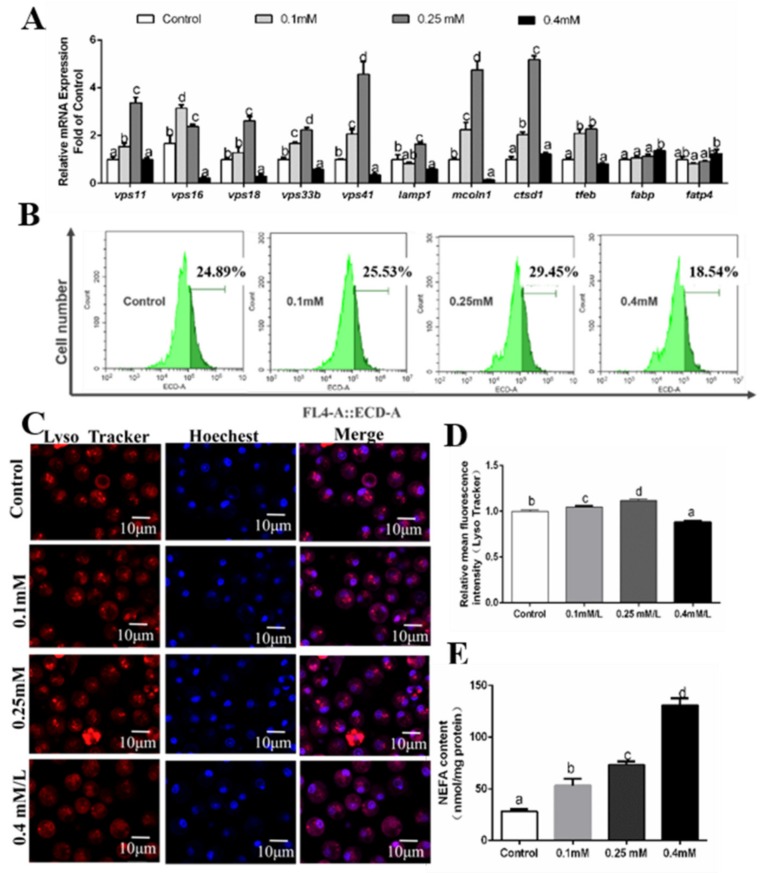
Effects of 48 h OA incubation on autophagosomes in hepatocytes of *P. fulvidraco.* (**A**) The effect of 48 h OA incubation on the mRNA levels of autolysosome-related genes; data (mean ± SEM, n = 3) were expressed relative to expression of housekeeping genes (*b2m* and *gapdh* (M = 0.1550)). (**B**) The effect of 48 h OA incubation on intracelluar autolysosomes demonstrated by flow cytometry analysis of the presence of Lyso Tracker-stained intracellular autolysosomes. (**C**) Representative confocal microscopy image of hepatocytes co-stained with Hoechest (blue) and Lyso Tracker (red) after 48 h OA incubation. (**D**) The effect of 48 h OA incubation on intracelluar autolysosomes demonstrated by flow cytometric analysis of red (FL4) mean fluorescence intensity. (**E**) Effects of 48 h OA incubation on NEFA content. Letters (*a–d*) denote significance, P < 0.05.

At 48 h, compared to the control, *vps11*, *ctsd1* and *tfeb* mRNA levels were higher in the 0.1 mM and 0.25 mM, but there were no significant differences for 0.4 mM OA group. mRNA levels of *vps16*, *vps18, vps33b, vps41* and *mcoln1* were higher in the 0.1 mM and 0.25 mM OA groups than in the control and lowest in the 0.4 mM OA group. *lamp1* mRNA expression was predominant in the control and 0.25 mM OA group. Compared to the control, OA addition up-regulated *fabp* and *fatp4* mRNA levels (Figure 8A). Intracellular NEFA content increased with OA concentration (Figure 8E). Lyso Tracker staining and flow cytometry analysis indicated that autolysosomes formation was induced when OA concentration increased from the control to 0.25 mM and then was inhibited at the 0.4 mM OA group (Figure 8B,D). Laser confocal microscope observation also confirmed this result (Figure 8C).

## 4. Discussion

The autophagy–lysosomal pathway was considered to play essential roles in maintaining metabolic homeostasis and regulating lipid metabolism [29], but whether fat and FAs can regulate the activity of the autophagosome–lysosome pathway remains unknown. Here, we identified nine genes involved in the autophagosome–lysosome pathway from *P. fulvidraco*, and explored their tissue distribution profiles and transcriptional responses to a high-fat diet. Moreover, we also explored the influences of PA and OA on their mRNA abundances and the formation of autolysosomes, which would contribute to our understanding of these genes’ function, and provide new insights into the mechanism of fat and FAs influencing lipid metabolism in vertebrates.

The elucidation of the structures of autophagy-related genes is significant for understanding their functional mechanisms. The present study indicated that all nine autophagosomal–lysosome pathway-related genes and proteins possessed conservative domains among multiple species [39,40,41], indicating that they share essential roles in common. For example, *P. fulvidraco vps11, vps18* and *vps41* possessed the conservative RING-H2 finger domain, which participated in protein/protein interactions [42,43]. The coiled coil domain in *P. fulvidraco* VPS16 was conserved in a variety of SNARE (soluble N-ethylmaleimide sensitive factor attachment protein receptor) proteins that mediated membrane docking and fusion pathways [44]. The *P. fulvidraco* VPS33B was cytosolic and contained a Sec1 domain, similar to several reports [34,35]. In fact, the HOPS complex, essential for homotypic vacuole fusion and vacuole protein sorting, consisted of VPS11, VPS33, VPS18, VPS16, VPS41 and VPS39 [8]. Thus, it appeared that the HOPS complex was conserved in similar vesicle-trafficking pathways between *P. fulvidraco* and other organisms. N-glycosylation is important for the protein stability in the lysosomal membrane [45], which was also observed in *P. fulvidraco* LAMP1. *P. fulvidraco* CTSD1 was composed of propeptide, signal peptide and a N-glycosylation site, similar to other reports [20,46]. These N-glycosylation sites were important for the transportation of cathepsins into lysosomes [47]. TFEB of *P. fulvidraco* contained HLH domain and LZ domain, similar to other reports [48]. The HLH and LZ domains have been shown to facilitate protein dimerization between different members of the same HLH or LZ family [48,49]. *P. fulvidraco* MCOLN1 showed similar architecture with mammals, containing six transmembrane domains, C-terminal cytoplasmic tails and channel pore. These results provided evidence that the autophagic process can be reconstituted in fish, which opened the possibility to further explore the molecular mechanism in the docking and fusion of autophagosomes and lysosomes in *P. fulvidraco*.

Our study determined their tissue distribution profiles in yellow catfish, which would provide some basic insights into their functions. Our results indicated that nine autolysosomess-related genes were widely detected in all sampled tissues, but their abundances were distinctly different, similar to other reports [20,23,44,46,50]. However, species–specific tissue distribution was also observed in yellow catfish. For example, yellow catfish *vps11* mRNA expression was predominant in the brain, muscle and spleen, then in the intestine, kidney, fat, heart, liver and lowest in the ovary. In contrast, Huizing et al. (2001) observed that *vps11* possessed a high expression in heart and pancreas in humans [41]. The expression of yellow catfish *ctsd1* mRNA was the highest in the kidney, then in the intestine, heart, spleen, fat, liver, brain and muscle, and lowest in the ovary. Liu et al. (2012) reported that the *ctsd1* transcripts were highest in the muscle, then in the liver and spleen, and low in other tissues [46]. To our best knowledge, for the first time we determined mRNA tissue expression of *vps33b, vps41 lamp1 mcoln1* and *tfeb* in fish. The observations in different tissue distribution in *P. fulvidraco* may represent the regulation of tissue specificity in physiology, indicating the different functions these issues play.

Our study clearly demonstrated that *vps11, vps16, vps18, vps33b, vps41, lamp1, mcoln1, ctsd1* and *tfeb* were negatively regulated at a transcriptional level by high-fat diets, indicating that the high dietary fat inhibited the fusion of autophagosomes and lysosomes. At present, the effects of dietary lipid levels on the response of autophagosomes and lysosomes are controversial. For example, Ebato et al. (2008) reported that high fat intake upregulated β-cell autophagy [51]. Koga et al. (2010) reported that a high-fat diet reduced autophagosome/lysosome fusion and downregulated autophagy [52]. These conflicting results are attributable to differences in cell types, experimental duration and dietary lipid levels. Our results supported that maintained lipid stimuli results in a primary defect in autophagosome/lysosome fusion, which could reinforce the importance of changes in intracellular lipid content on autophagic vesicular fusion.

FA-induced lipotoxicity plays critical roles in inducing the occurrence of non-alcoholic liver disease. However, the types of fatty acids influenced autophagy. Mei et al. (2011) found that saturated and unsaturated FAs regulated autophagy in a different manner. They pointed out that only OA but not PA induced autophagy [32]. The present study indicated that PA and OA incubation increased the concentration of intracellular NEFA in hepatocytes of yellow catfish. Moreover, our study for the first time indicated that PA and OA differentially influenced mRNA levels of the nine autophagosome–lysosome pathway-related genes and these effects seemed to be concentration- and time-dependent. Furthermore, flow cytometry results and laser confocal microscopy showed that PA addition significantly inhibited the formation of autolysosomes at 12 h, 24 h and 48 h. In contrast, OA incubation significantly inhibited the formation of autolysosomes at 24 h. At 12 h, OA significantly induced formation of autolysosomes, and at 48 h, autolysosomes formation was induced when OA concentration increased from the control to 0.25 mM and then inhibited at 0.40 mM OA group. Tan et al. (2012) pointed out that PA but not OA activated the autophagy [53]. On the other hand, Koga et al. (2010) reported that both PA and OA treatment inhibited autophagy by preventing the fusion of the autophagosome and lysosome [52]. Again, these conflicting results were attributable to the differences in the cell types, and the concentration and duration of FA treatment [53]^.^ Thus, elucidation of molecular mechanisms underlying the FA-induced autophagy awaits further investigation.

In summary, we identified the cDNA sequences of nine autolysosome-related genes from yellow catfish, detected their tissue expression and mRNA levels in yellow catfish fed a high-fat diet. Our results revealed the differential effects of saturated and unsaturated FAs on these genes’ expression. Further investigation should focus on the elucidation of molecular mechanisms during these processes.

## Figures and Tables

**Table 1:** The sequence information of autolysosome-related genes from *P. fulvidraco.*

Gene	Accession No.	5′ Untranslated Regions (UTR)(bp)	ORF (bp)	3′ UTR (bp)	Full Length (bp)	Protein (aa)
*vps11*	MH301091	22	2784	274	3080	928
*vps16*	MH301092	48	2508	589	3144	835
*vps18*	MH301093	305	2979	98	3379	992
*vps33b*	MH301094	69	1853	230	2230	617
*vps41*	MH301095	67	2564	427	2991	854
*lamp1*	MH301096	84	1245	167	1496	415
*mcoln1*	MH301097	217	1745	196	2158	581
*ctsd1*	MH301098	86	1190	569	1845	396
*tfeb*	MH459004	181	1475	367	2023	491

**Table 2:** Amino acid sequence identity of autolysosome-related genes between *P. fulvidraco* and other species (%).

Genes	*Ictalurus punctatus*	*Danio rerio*	*Xenopus tropicalis*	*Rattus norvegicus*	*Homo sapiens*
*vps11*	95.1	89.3	84.1	84.6	84.4
*vps16*	94.73	85.87	62.80	65.67	67.71
*vps18*	92.04	85.99	70.67	63.61	64.61
*vps33b*	93.19	88.01	75.12	74.64	63.65
*vps41*	96.02	91.80	77.52	80.09	80.56
*lamp1*	76.68	86.8	27.61	42.61	42.24
*mcoln1*	87.44	79.9	65.82	60.91	60.90
*ctsd1*	92.93	85.93	63.26	63.83	67.08
*tfeb*	86.60	69.82	44.20	48.65	46.55

Notes: Accession numbers for each gene are given with the species in the following order for each gene: The order is *Ictalurus punctatus*, *Danio rerio*, *Xenopus tropicalis*, *Rattus norvegicus* and *Homo sapiens*: *vps11* (AAI66363.1, NP_001032797.1, XP_017344206, 1AAI68871.1, AAH12051.2); *vps16* (XP_017336907.1, NP_001091659.1, AAH75508.1, NP_001005541.1, AAH73959.1); *vps18* (AHH37325.1, AAI54757.1, NP_001121454, 1AAI69083.1, AAH01513.1); *vps33b* (XP_017314277.1, NP_001014370.1, NP_001096504.2, NP_071622.1, CAB93109.1); *vps41* (AHH39036.1, XP_691671.2, XP_012814859.1, NP_001100825, NP_055211.2); *lamp1* (XP_017312814.1, NP_001313461.1, NP_001106388.1, NP_001101811.1, NP_003556.1); *mcoln1* (XP_017329106.1, NP_001299842.1, AAH80326.1, AAH61575.1, NP_003891.1); *ctsd1* (NP_001244039.1, AAI64814.1, AAH75272.1, NP_599161.2, CAG33228.1); *tfeb* (XP_017306305.1, NP_001244121.1, AAI23931.1, NP_001020878.1, NP_001161299.2).

## Supplementary Materials

The following are available online at https://www.mdpi.com/2073-4425/10/10/751/s1: Table S1: Nucleotide sequences of the primers used for the cDNA cloning from *P. fulvidraco*; Table S2. Feed formulation and proximate analysis of experimental diets; Table S3: Primers used for real-time quantitative PCR analysis; Figure S1: Multiple amino acid sequence alignment of VPS11 from *P. fulvidraco* and other species; Figure S2: Multiple amino acid sequence alignment of VPS16 from *P. fulvidraco* and other species; Figure S3: Multiple amino acid sequence alignment of VPS18 from *P. fulvidraco* and other species; Figure S4: Multiple amino acid sequence alignment of VPS33B from *P. fulvidraco* and other species; Figure S5. Multiple amino acid sequence alignment of VPS41 from *P. fulvidraco* and other species; Figure S6. Multiple amino acid sequence alignment of LAMP1 from *P. fulvidraco* and other species; Figure S7. Multiple amino acid sequence alignment of MCOLN1 from *P. fulvidraco* and other species; Figure S8. Accession numbers as follows: *P. fulvidraco* (Pf), MH301098; *Ictalurus punctatus* (Ip), NP_001244039.1; *Danio rerio* (Dr), AAI64814.1; *Rattus norvegicus* (Rn), NP_599161.2; *Homo sapiens* (Hs), CAG33228.1; Figure S9. Multiple amino acid sequence alignment of transcription factor EB (TFEB) from *P. fulvidraco* and other species; Figure S10. Phylogenetic tree based on the protein sequences of VPS11 from *P. fulvidraco* and other vertebrate species using the neighbor-joining (NJ) method in MEGA 5.0 based on the JTT+G model. Branch support values represent a percentage of 1000 bootstrap replicates; Figure S11. Phylogenetic tree based on the protein sequences of VPS16 from *P. fulvidraco* and other vertebrate species using the neighbor-joining (NJ) method in MEGA 5.0 based on the JTT+G model. Branch support values represent a percentage of 1000 bootstrap replicates; Figure S12. Phylogenetic tree based on the protein sequences of VPS18 from *P. fulvidraco* and other vertebrate species using the neighbor-joining (NJ) method in MEGA 5.0 based on the JTT+G model. Branch support values represent a percentage of 1000 bootstrap replicates; Figure S13. Phylogenetic tree based on the protein sequences of VPS33B from *P. fulvidraco* and other vertebrate species using the neighbor-joining (NJ) method in MEGA 5.0 based on the JTT+G model. Branch support values represent a percentage of 1000 bootstrap replicates; Figure S14. Phylogenetic tree based on the protein sequences of VPS41 from *P. fulvidraco* and other vertebrate species using the neighbor-joining (NJ) method in MEGA 5.0 based on the JTT+G model. Branch support values represent a percentage of 1000 bootstrap replicates; Figure S15. Phylogenetic tree based on the protein sequences of LAMP1 from *P. fulvidraco* and other vertebrate species using the neighbor-joining (NJ) method in MEGA 5.0 based on the JTT+G model. Branch support values represent a percentage of 1000 bootstrap replicates; Figure S16. Phylogenetic tree based on the protein sequences of MCOLN1 from *P. fulvidraco* and other vertebrate species using the neighbor-joining (NJ) method in MEGA 5.0 based on the JTT+G model. Branch support values represent a percentage of 1000 bootstrap replicates; Figure S17. Phylogenetic tree based on the protein sequences of CTSD1 from *P. fulvidraco* and other vertebrate species using the neighbor-joining (NJ) method in MEGA 5.0 based on the JTT+G model. Branch support values represent a percentage of 1000 bootstrap replicates; Figure S18. Phylogenetic tree based on the protein sequences of TFEB from *P. fulvidraco* and other vertebrate species using the neighbor-joining (NJ) method in MEGA 5.0 based on the JTT+G model Branch support values represent a percentage of 1000 bootstrap replicates.

## Abbreviations

b2m: beta-2-microglobulin; ctsd1, cathepsin D; elfa, translation elongation factor; fabp, fatty acid binding protein; fatp, fatty acid transport protein; gapdh, glyceraldehyde-3-phosphate dehydrogenase; hprt, hypoxanthine-guanine phosphoribosyltransferase; lamp1, lysosome-associated membrane glycoprotein1; mcoln1, mucolipin-1; OA: oleic acid; PA: palmitic acid; rpl7, ribosomal protein L7; SEM, standard error of mean; tuba, tubulin alpha chain; tfeb, transcription factor EB; ubce, ubiquitin-conjugating enzyme; vps, vacuolar protein sorting-associated protein.
