# Supplementary material for: Effects of Fat and Fatty Acids on the Formation of Autolysosomes in the Livers from Yellow Catfish Pelteobagrus Fulvidraco
Source: Genes (Basel). 2019 Sep 25;10(10):751. doi: 10.3390/genes10100751 (PMC6826758; doi:10.3390/genes10100751)
Supplement: Supplementary file 1 [file genes-10-00751-s001.pdf]

genes

MDPI

Supplemental Table S1. Nucleotide sequences of the primers used for the cDNA cloning from *P. fulvidraco*.

Primers	Sequences (5'-3')
	**Primers for partial fragment**
vps11 -F	CAACTBGACCGCCTNAAGARAAC
vps11-R	TGRTGAGBATGAACSTTAGTGCTT
vps16 -F	TTKCTCADACCTCCAHAATGTCGC
vps16 -R	GCCYTCTTGCYCCTCGTGKTCTAC
vps18-F	GCTHTGAGCGTCCSTCGGAGTA
vps18 -R	GGGTBTTGTGGTGCYTGGGGTTA
vps33b -F	GCCATGBCCMCGTTTGAGTTCCC
vps33b -R	CTTGHTGGTGDAGGCRGGTTGTG
vps41 -F	AGGATGRTGGAGADMGAGGATGG
vps41 -R	TGGGTGGTSTTCAVTGGTGAMTGTT
lamp1 -F	CTTHCTCBGCYGCTCCACAAACG
lamp1 -R	GCTCGRBGCHGTGAATCACCACT
mcoln1 -F	ACTGGTSCTGTTTCYCTGCTVTCTG
mcoln1 -R	GCYTGACGCHCTGGGTYTGAATA
ctsd1-F	CTGCYCATCCGTBTCCGTTTHCC
ctsd1 -R	CTGACGCACYCTCGTBCHCTCCC
tfeb-F	TCGCCTGTATGADGTTRTCCGAGT
tfeb -R	TAAAMGGGGTGATGASGGGACTG
**Primers for 3'-RACE PCR**	
3' GS-*vps11*-O	GGAGATCACCGAAGCACGACGCC
3' GS- *vps*11- I	AGGAGTACCCTAAAGTGGTCG
3' GS- *vps16* -O	TGATAAAGCAGACTGGGACGA
3' GS- *vps16* -I	CTGGCAGTAGGCGATTTGGAA
3' GS- *vps18*- O	GGACGAGTACCACCATCACAT
3' GS- *vps18* -I	CACCTGTGACTTTCCCTTACTC
3' GS- *vps33b* -O	AAGCACGAATCAAAACAGACC
3' GS- *vps33b* -I	GAGAAAGGCTATAAGTTTATCGTGG
3' GS- *vps41*-O	GCTGGGAAATGTCGATAAGGC
3' GS- *vps41* I	AAAGATGCACAGAACCCAGAT
3' GS- *lamp*1-O	CGGCACGACCTGTCTTCTT
3' GS- *lamp1* -I	TGAATCCACGACCGCTACT
3' GS-*mcoln1* -O	GCGTATGAGACCATCAAGCA
3' GS- *mcoln1* -I	TGACAGAACCACCACTTATGAA
3' GS- *ctsd1*-O	GCCTTCCCTTCCTACTATTTCC
3' GS- *ctsd1*-I	CACCTGTGACTTTCCCTTACTC
3' GS- *tfeb*-O	ATGGAGGAAGTTATTGAGGACA
3' GS- *tfeb*-I	ACTCCTGCCCTGCCAACCTACA
3' RACE Outer	TACCGTCGTTCCACTAGTGATTT
3' RACE Inner	CGCGGATCCTCCACTAGTGATTTCACTATAGG

**Primers for 5'-RACE PCR**	
5' GS- *vps11* -O	GAAGGCGATGAACTTATTGTCTAG
5' GS- *vps11* -I	CAAAAGCAAAACAAGGACCTCT
5' GS- *vps16* -O	GCTCATCGTCAGAAGAAAAGGCAATC
5' GS- *vps16*-I	TCAATGTTTGTTGCCAGGGTA
5' GS- *vps18*-O	GGCGTGGTCTTTGGGTTTCAGTG
5' GS- *vps18*-I	TCCAGCCTCACGTTCACAAAT
5' GS- *vps33b*-O	TCACATCACCGTAAACACCCT
5' GS- *vps33b*-I	GCCTATGAGGTCCTTCCTGCTGA
5' GS- *vps41*-O	GGTGTTTGTTTCGTTGCCTAA
5' GS- *vps41-I*	GTCTTCTTCCTCCGAGTCATC
5' GS- *lamp1* -O	GGGAAACTCCTGCTTCACC
5' GS *lamp1* -I	CTCGTGCCATTGATGATAGG
5' GS- *mcoln1*-O	GCTTTCTTCCCTTGGCATAGTA
5' GS- *mcoln1*-I	TTTGCCGTCATGCGAACCATA
5' GS- *ctsd1*-O	GCCCCATTATACTTGTGATGAAGC
5' GS- *ctsd1*-I	TCTCACCATAGTATTGGGCATC
5' GS- *tfeb-O*	TTCTGAGACTGGCGGATGTGG
5' GS-*tfeb*- I	TGTTTGTACCTTCAGCACCTC
5' RACE Outer	TAGGGCCTAATACGACTCACTA
5' RACE Inner	CTAATACGACTCACTATAGGGCA AGCAGTGGTATCAACGCAGAGT

Notes: K-G/T; M-A/C; R-A/G; S-G/T; Y-C/T; B-G/T/C; D-G/A/T; H-A/T/C; V-G/A/C; N-A/T/G/C. **Abbreviations:** *ctsd1*; Cathepsin D; *lamp*; lysosome-associated membrane glycoprotein1; *mcoln1*: mucolipin-1; *tfeb*: transcription factor EB; *vps,* vacuolar protein sorting-associated protein.

Supplemental Table S2. Primers used for real-time quantitative PCR analysis.

Genes	Forward primer (5′-3′)	Reverse primer (5′-3′)	Size	Accession no.
vps11	GCAGAGAGTGAGGCTGAGTG	AATCTGCCACCACCGAGAAG	150	MH301091
vps16	TGTCCTGTCTCACTGTTCGC	CCTGCAGGTTCCGTCTCATT	184	MH301092
vps18	CCGGAGAGAAGACGCAGATC	GGTGCTCCGGTTGTTGTAGA	182	MH301093
vps33b	TTGGGCCTGAGGTCACTTC	AGACGGTGCTCCTGTTTCAG	230	MH301094
vps41	TCTCACTGTCCGCAACTTCC	TTGTCAAGCAGCCAGTCGAT	153	MH301095
lamp1	CAGCAACAGCAGTAGTGGGA	AGCTCAGTGTAAGGTTGGCC	150	MH301096
mcoln1	ATCGTGACCCAAACTCGCTT	GGTCGGCGGGTATATCTTGG	184	MH301097
ctsd1	TTTGTAAGGGAGGCTGCGAG	TGTCCGCCCAAATTGAAGGA	150	MH301098
tfeb	ACCAGCGACCTCCTCCTAAT	AGCTCAAATCTCCCAGGCAC	240	MH459004
β-actin	GGACTCTGGTGATGGTGTGA	CTGTAGCCTCTCTCGGTCAG	138	EU161066
rpl7	GGCAAATGTACAGGAGCGAG	GCCTTGTTGAGCTTGACGAA	199	KP938522
hprt	ATGCTTCTGACCTGGAACGT	TTGCGGTTCAGTGCTTTGAT	181	KP938523
tuba	TCAAAGCTGGAGTTCTCGGT	AATGGCCTCGTTATCCACCA	135	KP938526
b2m	GCTGATCTGCCATGTGAGTG	TGTCTGACACTGCAGCTGTA	186	KP938520
ubce	TCAAGAAGAGCCAGTGGAGG	TAGGGGTAGTCGATGGGGAA	150	KP938524
tbp	AGCAAAGAGTGAGGAGCAGT	ACTGCTGATGGGTGAGAACA	170	KP938525
gapdh	TTTCAGCGAGAGAGACCCAG	ATGACTCTCTTGGCACCTCC	132	KP938521
18s rRNA	AGCTCGTAGTTGGATCTCGG	CGGGTATTCAGGCGAGTTTG	196	KP938527
elfa	GTCTGGAGATGCTGCCATTG	AGCCTTCTTCTCAACGCTCT	151	KU886307
fatp4	TCGTGGTGGTGAGGACTTTG	CATTGAGCAAGACGGAAAC A	164	MG637279
fabp	TGCCCCTCACATAGT TGCTG	CACTTCCTCGAACA TCCCTCAT	148	MG882758

Abbreviations: *b2m*, beta-2-microglobulin *ctsd1*; Cathepsin D1; *elfa*, translation elongation factor; *gapdh*, Glyceraldehyde-3-phosphate dehydrogenase; *hprt*, hypoxanthine-guanine phosphoribosyl transferase; *lamp1*; lysosome-associated membrane glycoprotein1; *mcoln1*: mucolipin-1; *rpl7*, ribosomal protein L7; *tuba*, tubulin alpha chain; *tfeb*: transcription factor EB; *ubce*, ubiquitin-conjugating enzyme; *vps*, vacuolar protein sorting-associated protein; *fabp*, fatty acid binding protein;

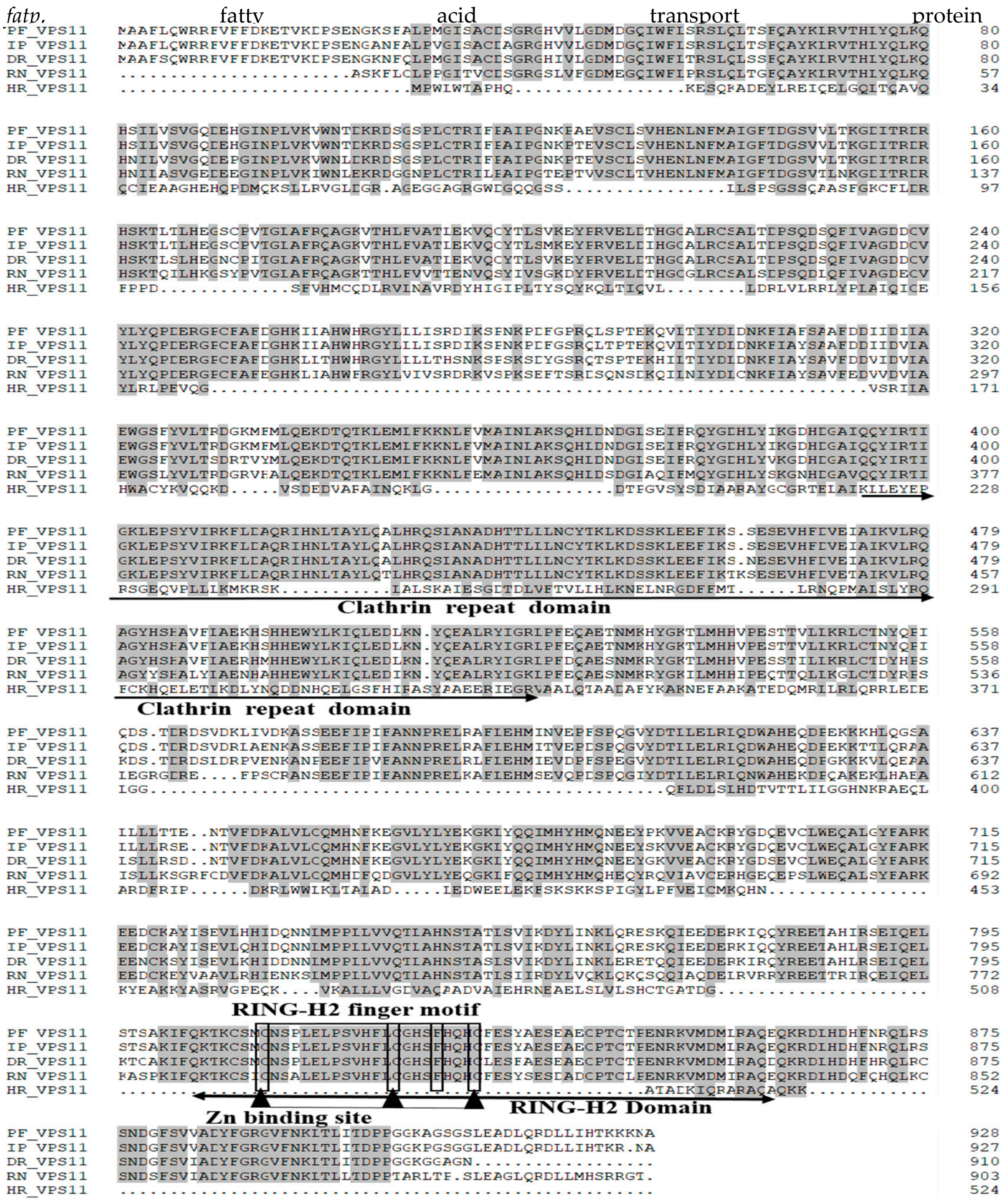

Supplemental Figure 1. Multiple amino acid sequence alignment of VPS11 from *P. fulvidraco* and other species. Accession numbers as followed: *Pelteobagrus fulvidraco* (Pf), MH301091; *Ictalurus punctatus* (Ip), AAI66363.1; *Danio rerio* (Dr), NP_001032797.1; *Rattus norvegicus* (Rn), 1AAI68871.1; *Homo sapiens* (Hs) AAH12051.2). Arrows below the sequences represented Clathrin repeat domain and RING-H2 domain. RING-H2 finger motif were boxed. Zn binding site were marked with the symbol (▲).

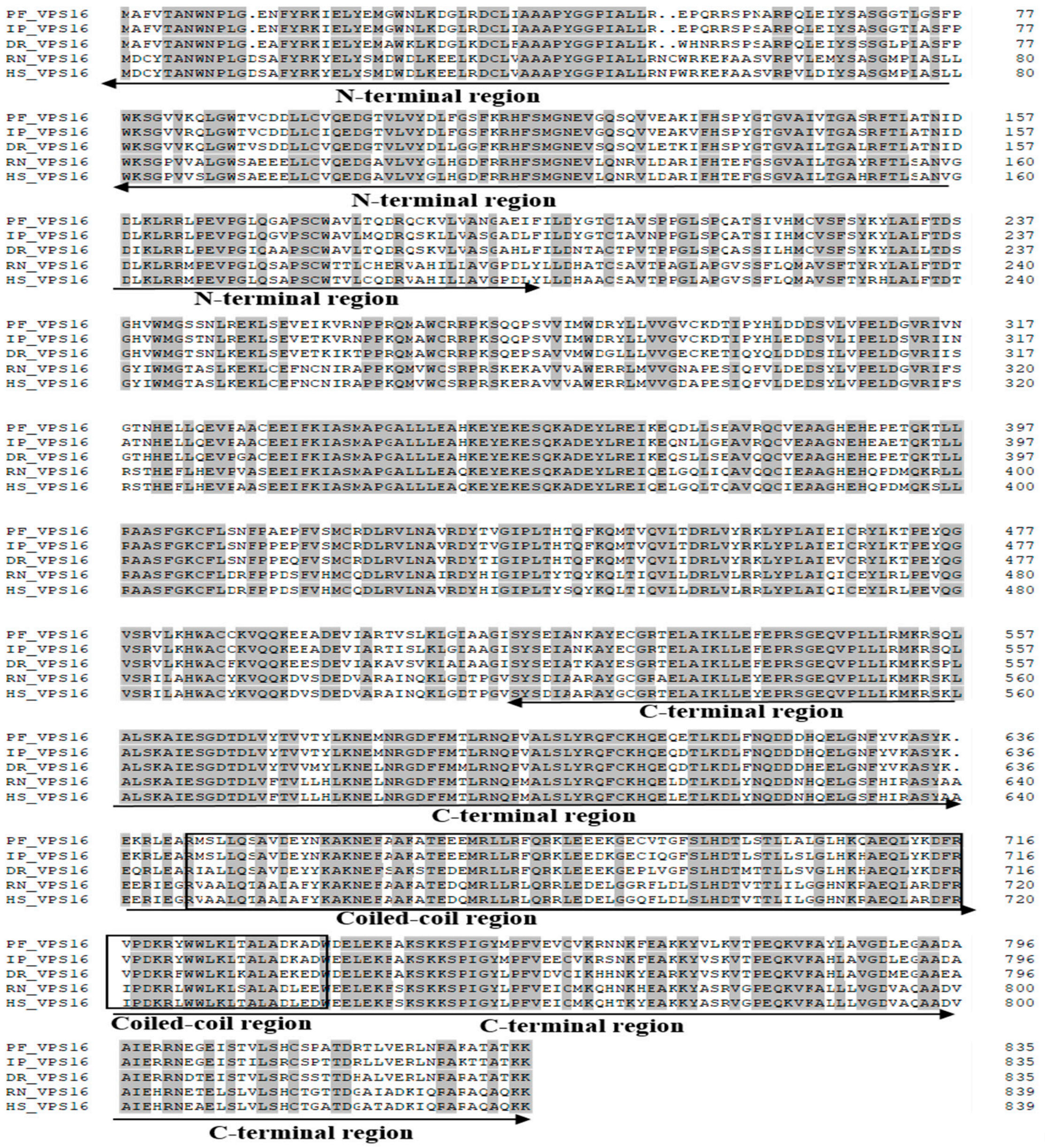

Supplemental Figure 2. Multiple amino acid sequence alignment of VPS16 from *P. fulvidraco* and other species. Accession numbers as followed: *Pelteobagrus fulvidraco* (Pf), MH301092; *Ictalurus punctatus* (Ip), XP_017336907.1; *Danio rerio* (Dr), NP_001091659.1; *Rattus norvegicus* (Rn), NP_001005541.1; *Homo sapiens* (Hs), AAH73959.1). Arrows below the sequences represented conserved domains: N-terminal region and C-terminal region. Coiled coil domain was boxed.

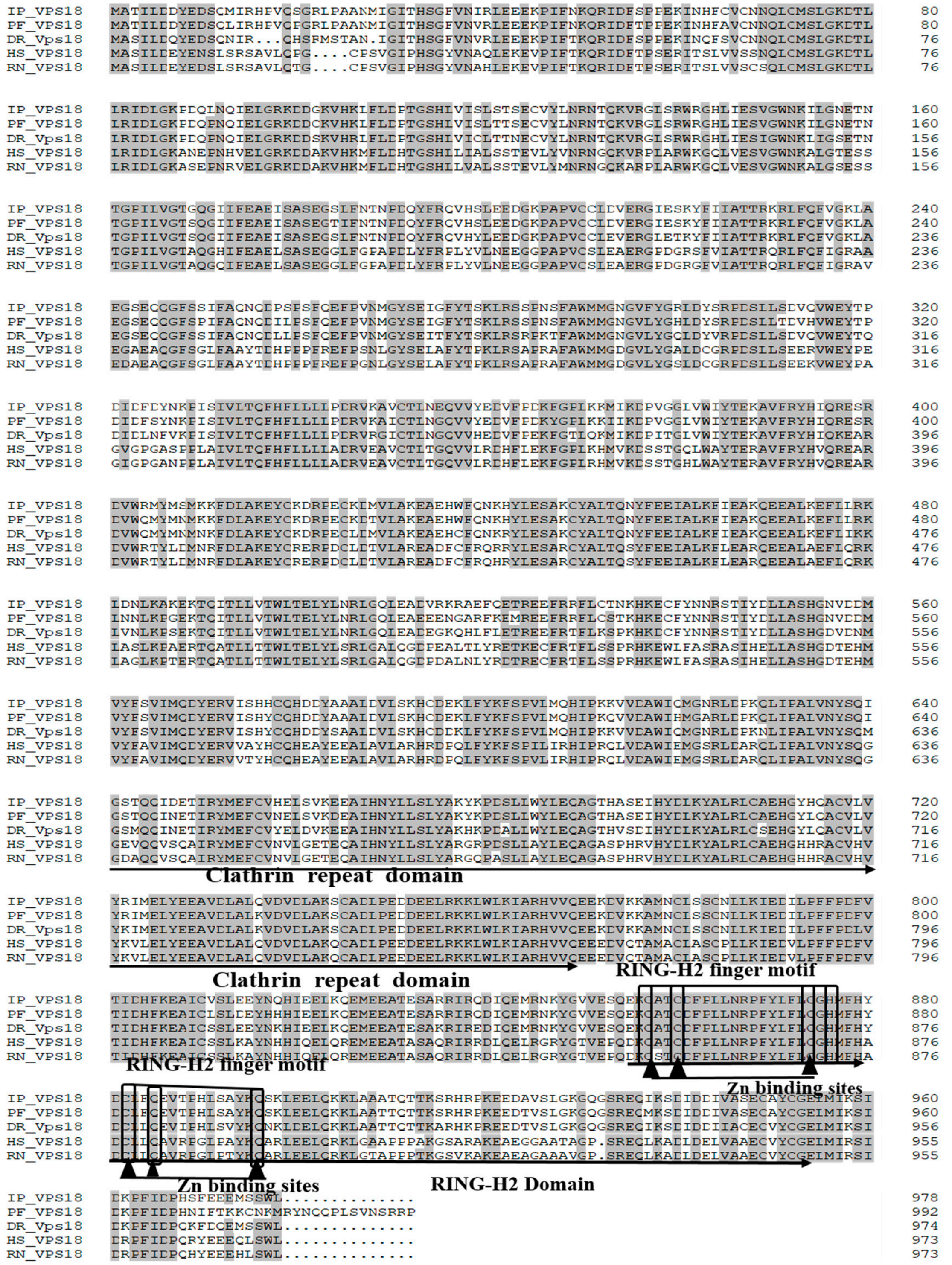

Supplemental Figure 3. Multiple amino acid sequence alignment of VPS18 from *P. fulvidraco* and other species. Accession numbers (*Pelteobagrus fulvidraco* (Pf) VPS18, MH301093; *Ictalurus punctatus* (Ip) VPS18, AHH37325.1; *Danio rerio* (Dr) VPS18, 1, AAI54757.1; *Rattus norvegicus* (Rn) VPS18, 1AAI69083.1; *Homo sapiens* (Hs) VPS18, AAH01513.1). Arrows below the sequences represented conserved Domain: Clathrin repeat domain. The RING-H2 domains were boxed, and the six Zn binding site were marked with the symbol (▲).

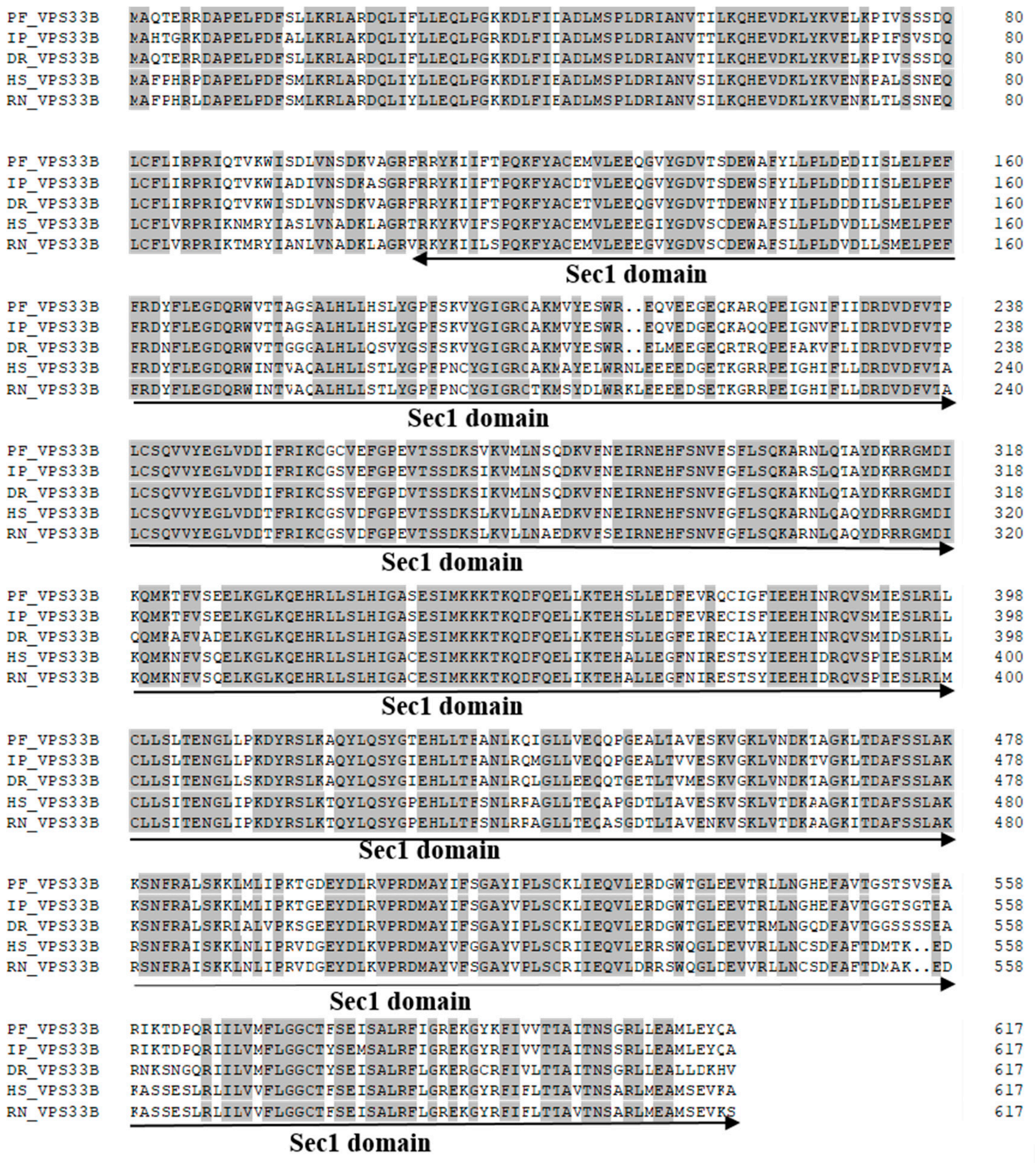

Supplemental Figure 4. Multiple amino acid sequence alignment of VPS33B from *P. fulvidraco* and other species. Accession numbers as follows: *P. fulvidraco* (Pf), MH301094; *Ictalurus punctatus* (Ip), XP_017314277.1; *Danio rerio* (Dr), NP_001014370.1; *Rattus norvegicus* (Rn) NP_071622.1; *Homo sapens*

(Hs), CAB93109. Arrows below the sequences represented conserved Sec1 domain.

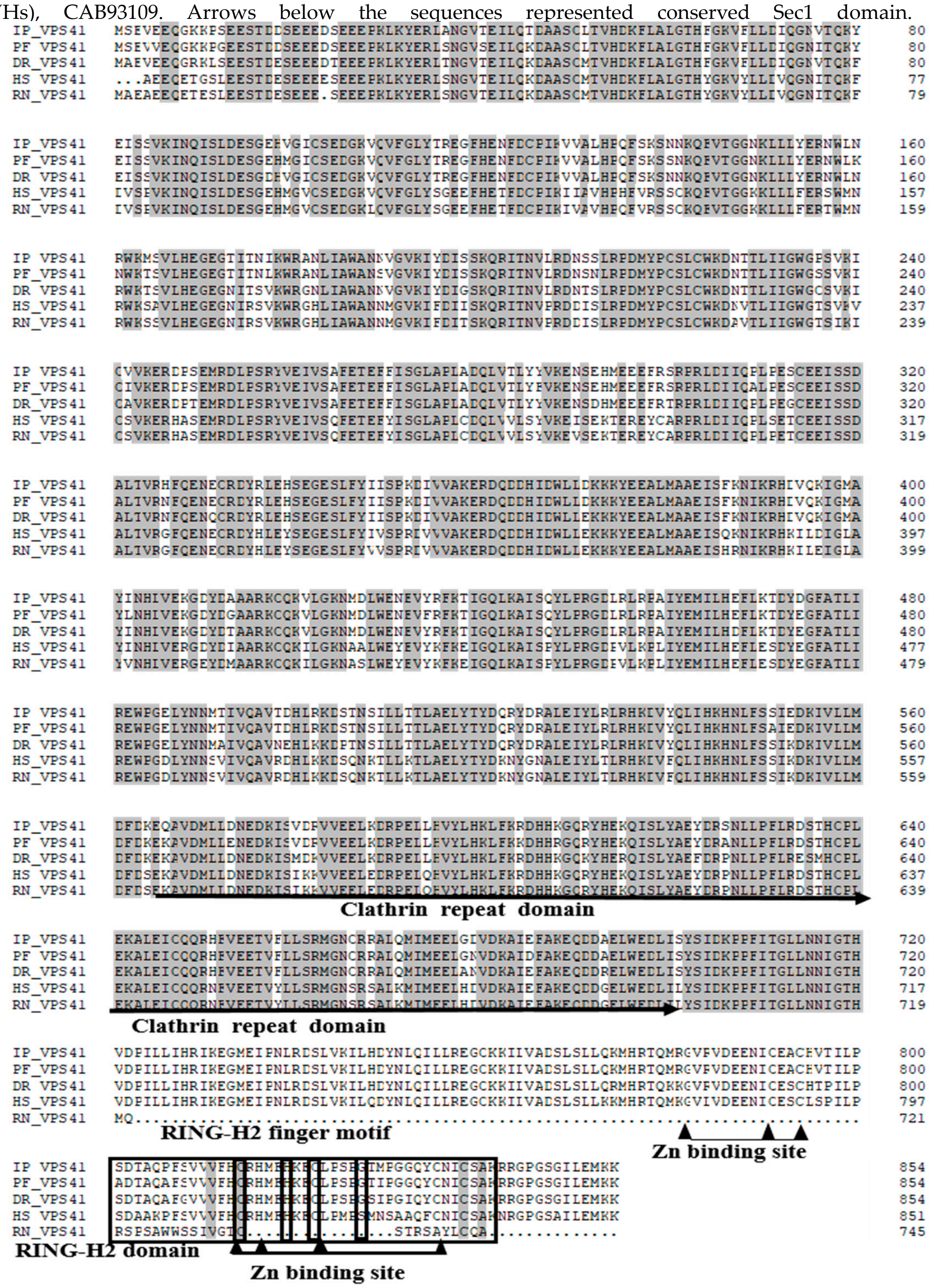

Supplemental Figure 5. Multiple amino acid sequence alignment of VPS41 from *P. fulvidraco* and other species. Accession numbers as followed: *P. fulvidraco* (Pf), MH301095; *Ictalurus punctatus* (Ip), AHH39036.1; *Danio rerio* (Dr), XP_691671.2; *Rattus norvegicus* (Rn), NP_001100825; *Homo sapiens* (Hs), NP_055211.2. Arrows below the sequences represented the conserved domains: Clathrin repeat domain and RING-H2 domain. The RING-H2 domain were boxed; The Zn binding site was marked with the symbol (▲).

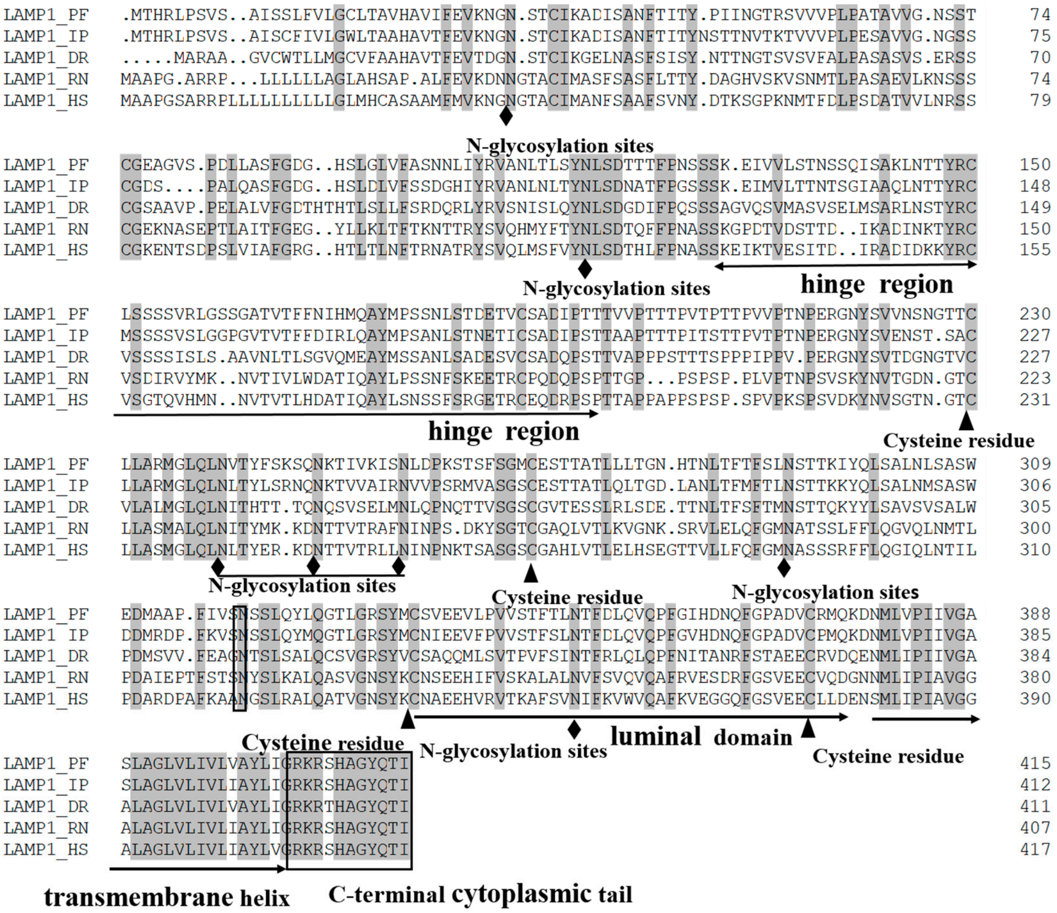

Supplemental Figure 6. Multiple amino acid sequence alignment of LAMP1 from *P. fulvidraco* and other species. Accession numbers as followed: *P. fulvidraco* (Pf), MH301096; *Ictalurus punctatus* (Ip), XP_017312814.1; *Danio rerio* (Dr), NP_001313461.1; *Rattus norvegicus* (Rn), NP_003556.1; *Homo sapiens* (Hs), NP003556.1. Arrows below the sequences represented conserved domains: luminal domain, one transmembrane helix domain and hinge region. The C-terminal cytoplasmic tail were boxed, and the cysteine residue was marked with the symbol (▲), and N-glycosylation was marked with the symbol (◆).

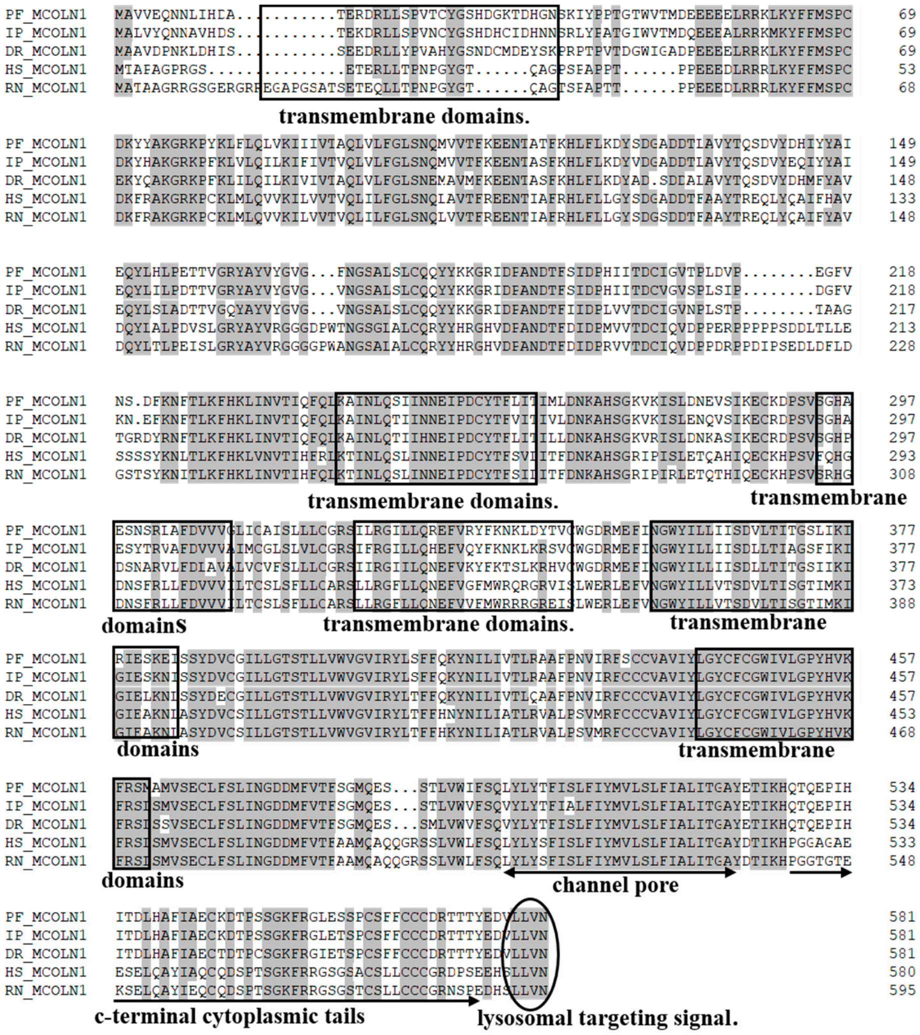

Supplemental Figure 7. Multiple amino acid sequence alignment of MCOLN1 from *P. fulvidraco* and other species. Accession numbers as follows: *P. fulvidraco* (Pf), MH301097; *Ictalurus punctatus* (Ip), XP017329106.1; *Danio rerio* (Dr), NP001299842.1; *Rattus norvegicus* (Rn), AAH61575.1; *Homo sapiens* (Hs), NP003891.1. Arrows below the sequences represented conserved domain: C-terminal cytoplasmic tails and channel pore. Six transmembrane domains were boxed, and lysosomal targeting signal was marked with circles.

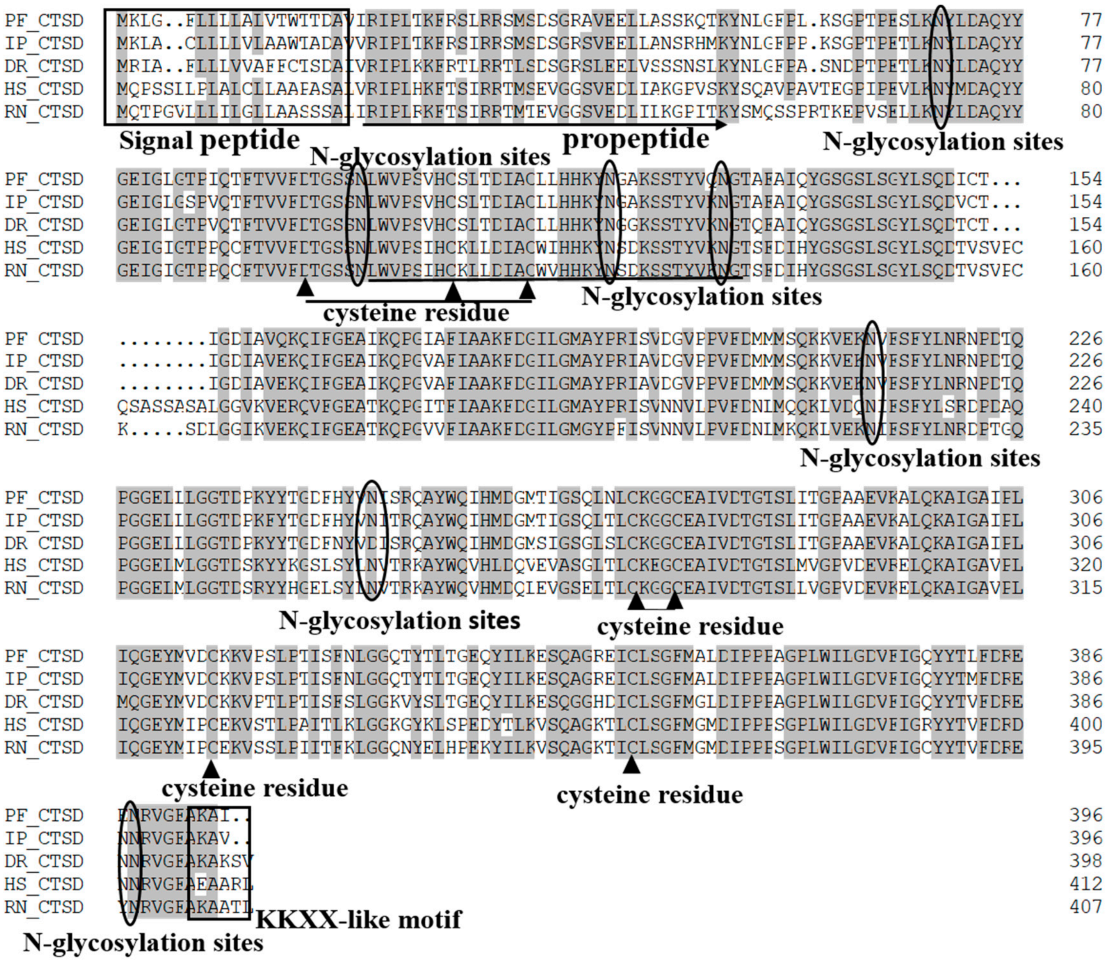

Supplemental Figure 8. Multiple amino acid sequence alignment of *ctsd1* from *P. fulvidraco* and other species. Accession numbers as follows: *P. fulvidraco* (Pf), MH301098; *Ictalurus punctatus* (Ip), NP_001244039.1; *Danio rerio* (Dr), AAI64814.1; *Rattus norvegicus* (Rn), NP_599161.2; *Homo sapiens* (Hs), CAG33228.1. Arrows below the sequences represented the conserved domain of the propeptide. Signal peptide and kkxx-like motif was boxed; N-glycosylation was circled, and cysteine residue site was marked with the symbol (▲).

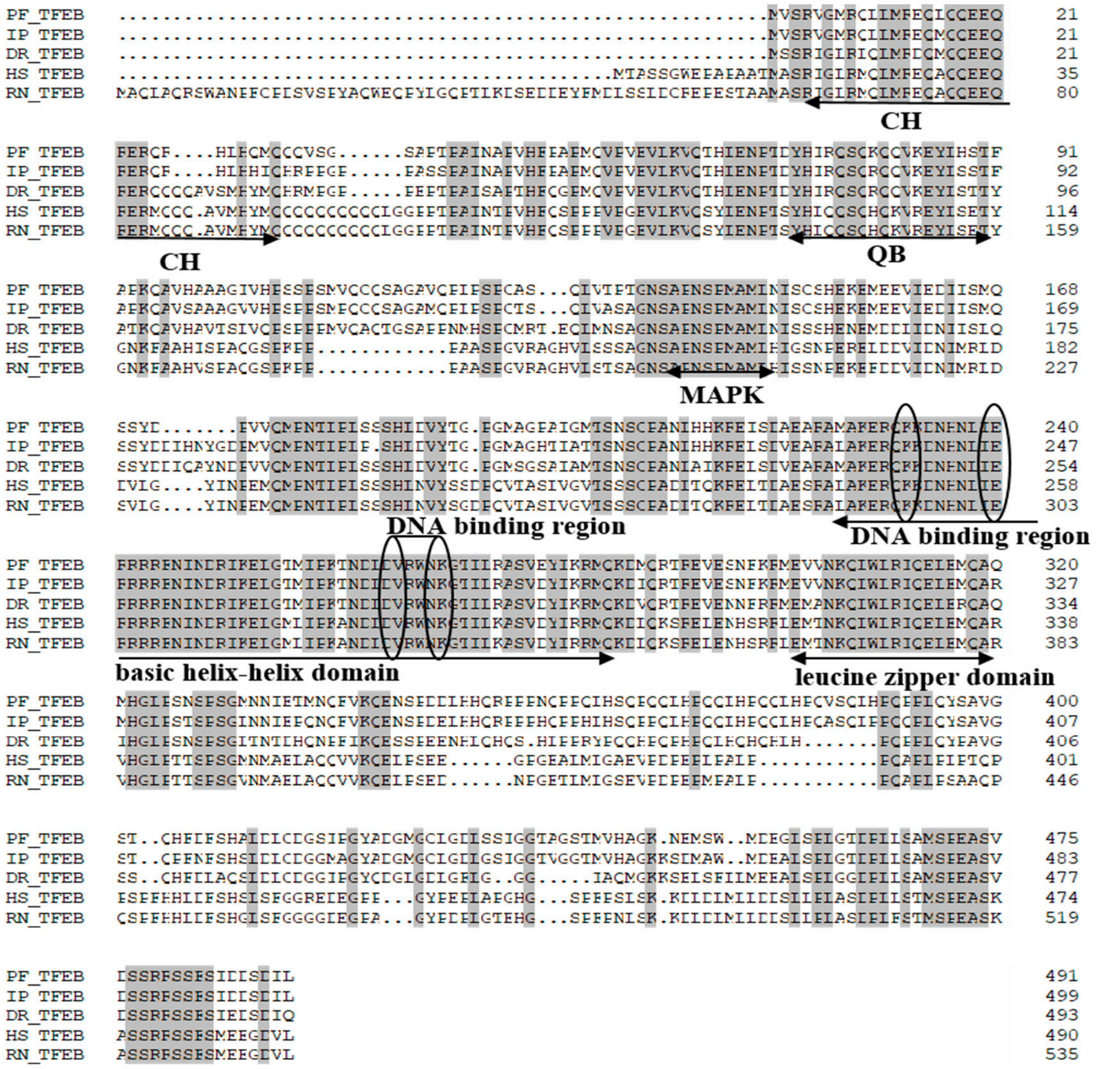

Supplemental Figure 9. Multiple amino acid sequence alignment of TFEB from *P. fulvidraco* and other species. Accession numbers as follows: *Pelteobagrus fulvidraco* (Pf), MH459004; *Ictalurus punctatus* (Ip), XP_017306305.1; *Danio rerio* (Dr), NP_001244121.1; *Rattus norvegicus* (Rn), NP_001020878.1; *Homo sapiens* (Hs), NP_001161299.2. Arrows below the sequences represented conserved domains: basic region helix-loop helix domain and the leucine zipper. DNA bind site was circled. Abbreviations: CH, charged helical domain; QB, glutamine-rich, basic domain; MAPK, consensus MAP kinase phosphorylation site.

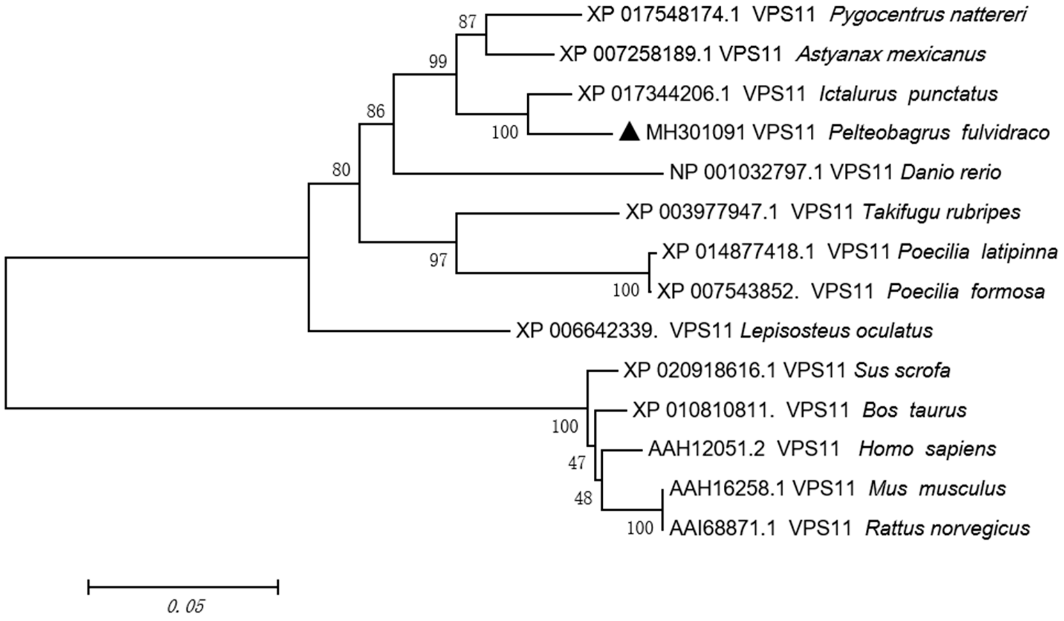

Supplemental Figure 10. Phylogenetic tree based on the protein sequences of VPS11 from *P. fulvidraco* and other vertebrate species using the neighbor-joining (NJ) method in MEGA 5.0 [(33)] based on the JTT+G model [(34)]. Branch support values represented a percentage of 1000 bootstrap replicates.

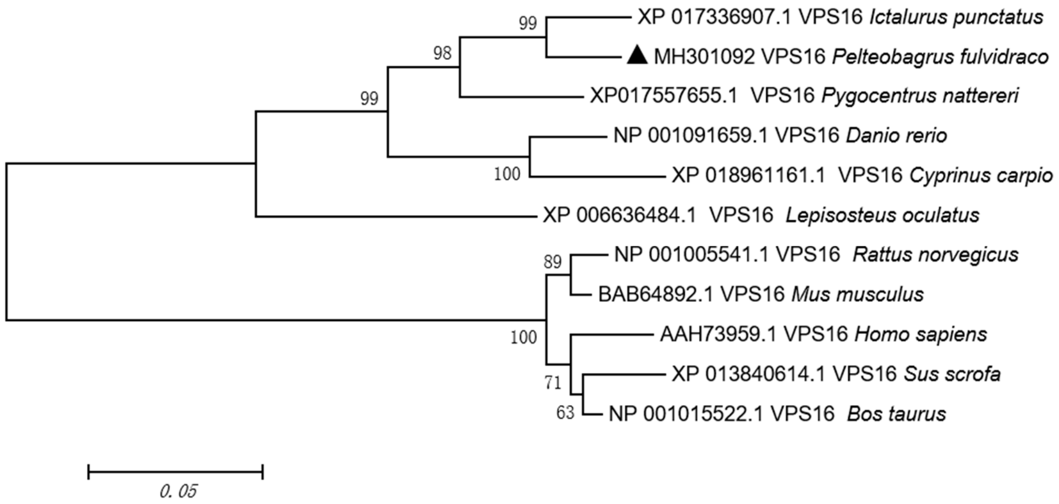

Supplemental Figure 11. Phylogenetic tree based on the protein sequences of VPS16 from *P. fulvidraco* and other vertebrate species using the neighbor-joining (NJ) method in MEGA 5.0 [(33)] based on the JTT+G model [(34)]. Branch support values represented a percentage of 1000 bootstrap replicates.

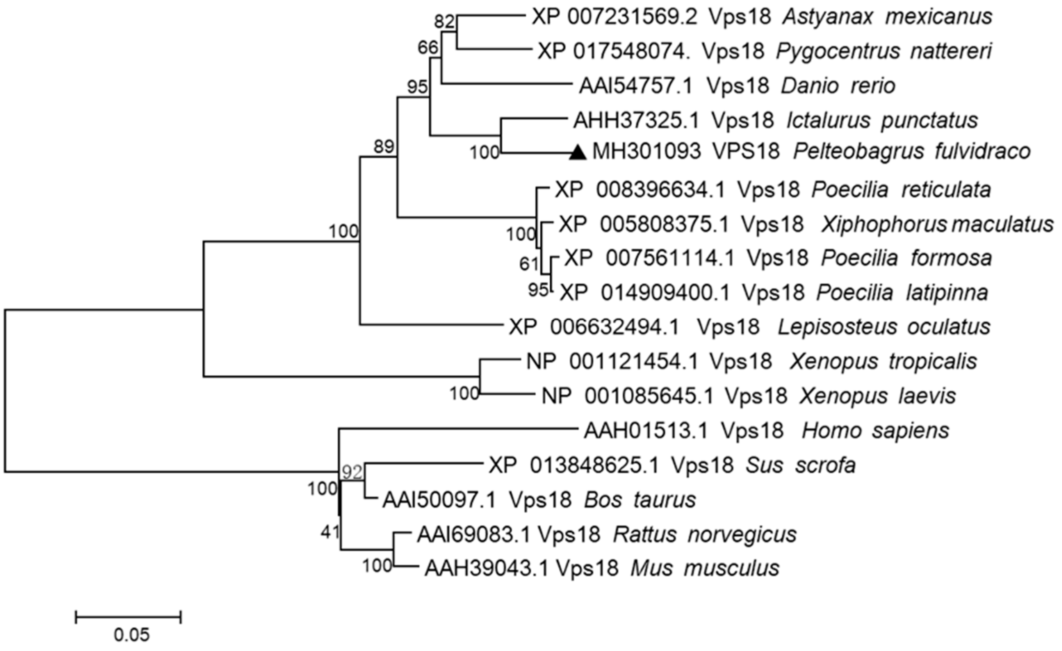

Supplemental Figure 12. Phylogenetic tree based on the protein sequences of VPS18 from *P. fulvidraco* and other vertebrate species using the neighbor-joining (NJ) method in MEGA 5.0 [33] based on the JTT+G model [34]. Branch support values represented a percentage of 1000 bootstrap replicates.

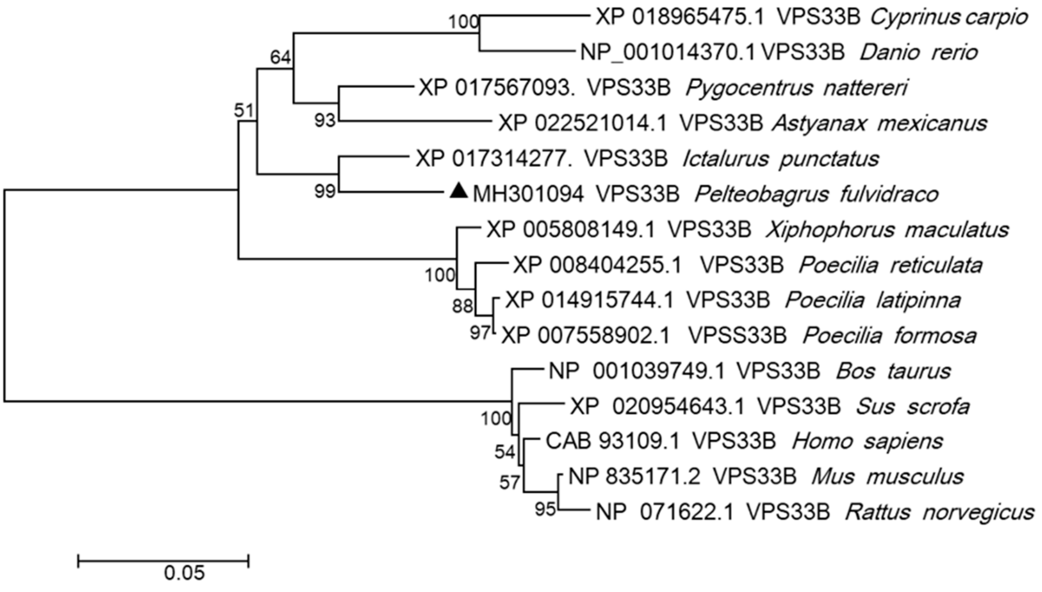

Supplemental Figure 13. Phylogenetic tree based on the protein sequences of VPS33B from *P. fulvidraco* and other vertebrate species using the neighbor-joining (NJ) method in MEGA 5.0 [33] based on the JTT+G model [34]. Branch support values represented a percentage of 1000 bootstrap replicates.

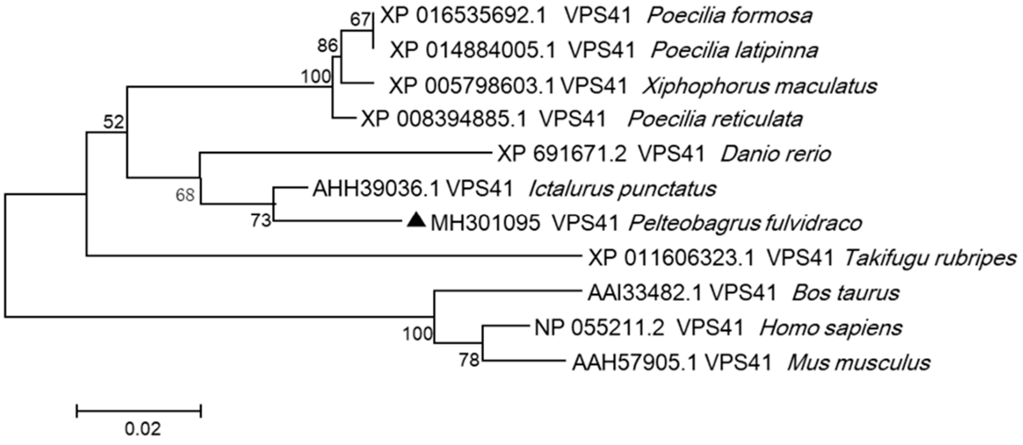

Supplemental Figure 14. Phylogenetic tree based on the protein sequences of VPS41 from *P. fulvidraco* and other vertebrate species using the neighbor-joining (NJ) method in MEGA 5.0 [(33)] based on the JTT+G model [(34)]. Branch support values represented a percentage of 1000 bootstrap replicates.

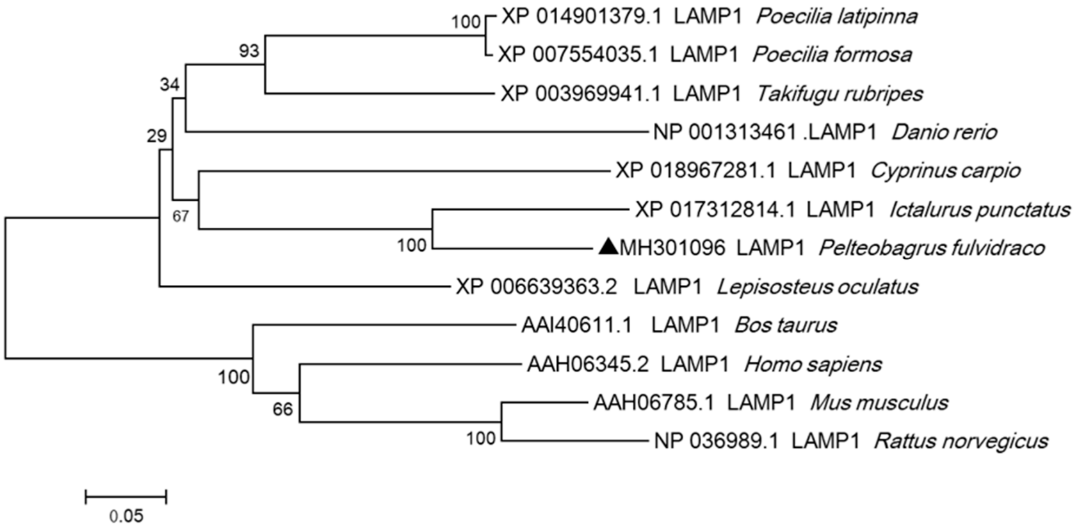

Supplemental Figure 15. Phylogenetic tree based on the protein sequences of LAMP1 from *P. fulvidraco* and other vertebrate species using the neighbor-joining (NJ) method in MEGA 5.0 [(33)] based on the JTT+G model [(34)]. Branch support values represented a percentage of 1000 bootstrap replicates.

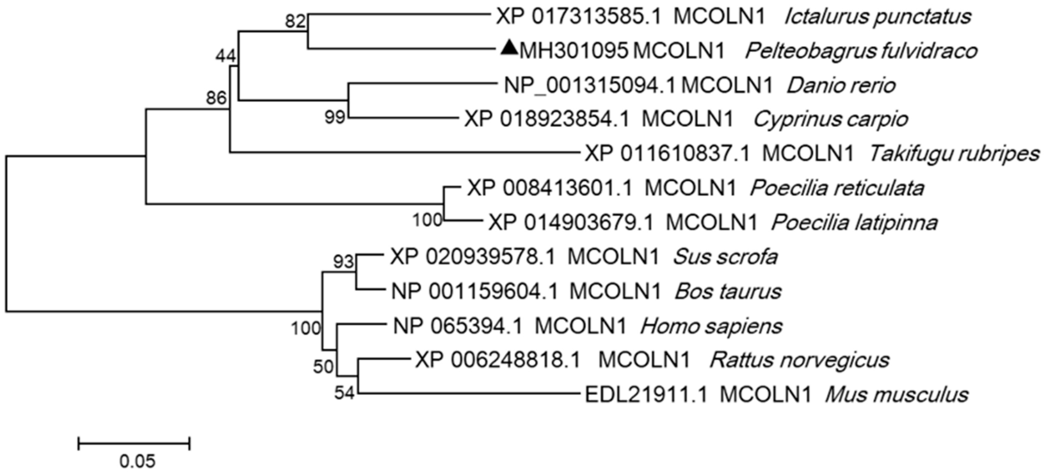

Supplemental Figure 16. Phylogenetic tree based on the protein sequences of MCOLN1 from *P. fulvidraco* and other vertebrate species using the neighbor-joining (NJ) method in MEGA 5.0 [33] based on the JTT+G model [34]. Branch support values represented a percentage of 1000 bootstrap replicates.

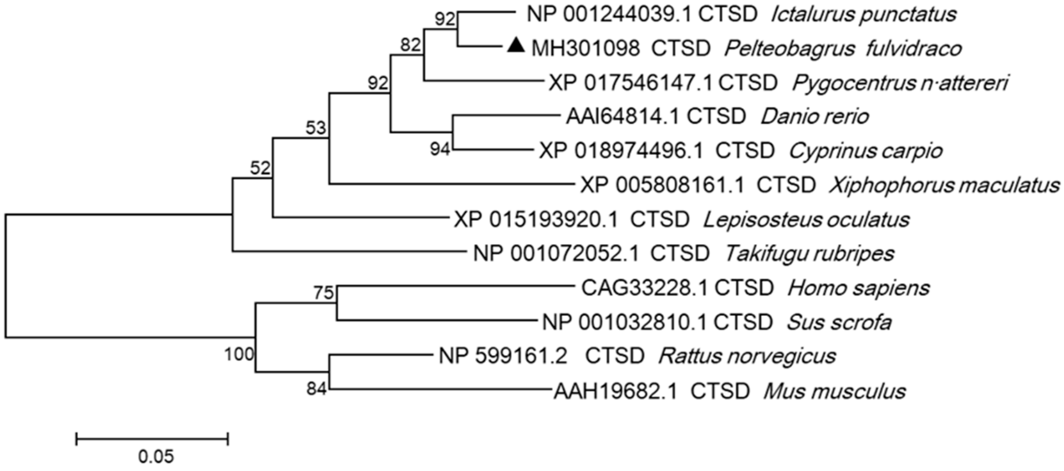

Supplemental Figure 17. Phylogenetic tree based on the protein sequences of CTSD1 from *P. fulvidraco* and other vertebrate species using the neighbor-joining (NJ) method in MEGA 5.0 [33] based on the JTT+G model [34]. Branch support values represented a percentage of 1000 bootstrap replicates.

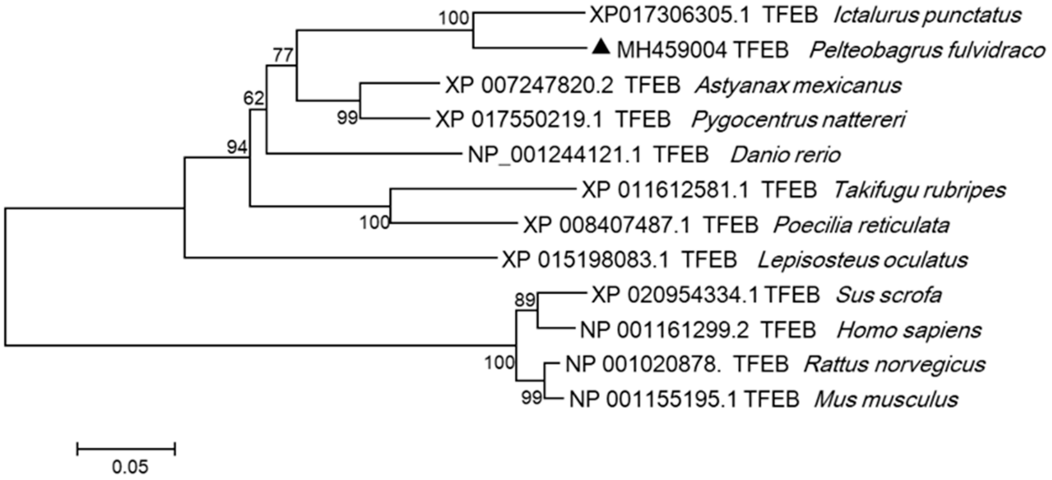

Supplemental Figure 18. Phylogenetic tree based on the protein sequences of TFEB from *P. fulvidraco* and other vertebrate species using the neighbor-joining (NJ) method in MEGA 5.0 [33] based on the JTT+G model [34]. Branch support values represented a percentage of 1000 bootstrap replicates.

© 2019 by the authors. Licensee MDPI, Basel, Switzerland. This article is an open access article distributed under the terms and conditions of the Creative Commons Attribution (CC BY) license (http://creativecommons.org/licenses/by/4.0/).
